# Dynamic response of RNA editing to temperature in *Drosophila*

**DOI:** 10.1186/s12915-014-0111-3

**Published:** 2015-01-03

**Authors:** Leila E Rieder, Yiannis A Savva, Matthew A Reyna, Yao-Jen Chang, Jacquelyn S Dorsky, Ali Rezaei, Robert A Reenan

**Affiliations:** Department of Molecular Biology, Cellular Biology, and Biochemistry, Brown University, Providence, RI 02912 USA; Department of Mathematical Sciences, Rensselaer Polytechnic Institute, Troy, NY 12180 USA

**Keywords:** ADAR, RNA editing, RNA thermometers, Temperature, Editosome, Poikilotherm, Inosine, A-to-I editing

## Abstract

**Background:**

Adenosine-to-inosine RNA editing is a highly conserved process that post-transcriptionally modifies mRNA, generating proteomic diversity, particularly within the nervous system of metazoans. Transcripts encoding proteins involved in neurotransmission predominate as targets of such modifications. Previous reports suggest that RNA editing is responsive to environmental inputs in the form of temperature alterations. However, the molecular determinants underlying temperature-dependent RNA editing responses are not well understood.

**Results:**

Using the poikilotherm *Drosophila*, we show that acute temperature alterations within a normal physiological range result in substantial changes in RNA editing levels. Our examination of particular sites reveals diversity in the patterns with which editing responds to temperature, and these patterns are conserved across five species of *Drosophilidae* representing over 10 million years of divergence*.* In addition, we show that expression of the editing enzyme, ADAR (adenosine deaminase acting on RNA), is dramatically decreased at elevated temperatures, partially, but not fully, explaining some target responses to temperature. Interestingly, this reduction in editing enzyme levels at elevated temperature is only partially reversed by a return to lower temperatures. Lastly, we show that engineered structural variants of the most temperature-sensitive editing site, in a sodium channel transcript, perturb thermal responsiveness in RNA editing profile for a particular RNA structure.

**Conclusions:**

Our results suggest that the RNA editing process responds to temperature alterations via two distinct molecular mechanisms: through intrinsic thermo-sensitivity of the RNA structures that direct editing, and due to temperature sensitive expression or stability of the RNA editing enzyme. Environmental cues, in this case temperature, rapidly reprogram the *Drosophila* transcriptome through RNA editing, presumably resulting in altered proteomic ratios of edited and unedited proteins.

**Electronic supplementary material:**

The online version of this article (doi:10.1186/s12915-014-0111-3) contains supplementary material, which is available to authorized users.

## Background

Natural DNAs are usually limited to double-stranded helical shapes; however, RNA is different — the repertoire of possible RNA secondary and tertiary structures appears limitless. RNA secondary structure is strongly correlated with function, and both the structure and corresponding thermodynamic stability of an RNA molecule contribute to functional regulation [1]. Dynamic RNA structures are acutely responsive to input in the form of molecular and environmental factors; it is this mutability of RNA structure that allows RNA to act as a sensor and elicit rapid cellular responses [2].

RNA structure is fundamentally sensitive to abiotic factors, such as temperature and metal ion concentration. Bacterial RNA thermometers, riboswitches sensitive to temperature, are responsive regulatory elements that control translation of heat-shock, cold-shock, and virulence genes [3,4]. Yet, there is no direct evidence of translational RNA thermometers in eukaryotes. With the addition of large expanses of intronic sequence, eukaryotic RNA thermometers could be considerably less conserved than those found in bacteria, confounding detection. Indeed, the regulation of alternative splicing by the eukaryotic thymidine pyrophosphate riboswitch depends on complex long-distance nucleotide interactions [5]. Therefore, temperature-sensitive structures found in eukaryotic mRNA could, in theory, act anywhere in the transcript to alter processing, translation, transport, degradation or protein binding.

Adenosine-to-inosine (A-to-I) RNA editing is a post-transcriptional modification known to be directed by secondary [6] and tertiary RNA structures, including those that act over a distance of up to several thousand nucleotides [7,8]. We reasoned that there might be a class of eukaryotic RNA thermometer-like structures that, instead of controlling translation, rather exert their effects on RNA editing levels. A-to-I RNA editing involves the hydrolytic deamination of adenosine into inosine, which is read as guanosine by the protein synthesis machinery. Editing, therefore, has the ability to recode the transcriptome at select sites [9] and diversify the proteome.

ADARs (adenosine deaminases acting on RNA), the highly conserved proteins responsible for A-to-I editing in all metazoa, are found localized to both cytoplasm and nucleus. While there are multiple ADAR proteins in mammals, the mammalian ADAR2 and single *Drosophila* ADAR (dADAR) appear to function primarily in the neuronal nucleus [10]. This is consistent with the observation that ADAR’s target transcripts encode proteins involved in chemical and electrical neurotransmission. Phenotypes in model organisms with editing deficiencies range from embryonic lethality [11] and seizures [12] (mouse) to defects in motor control [13] (*Drosophila*) and chemotaxis [14] (*Caenorhabditis elegans*). Such phenotypic consequences of ADAR deficiency indicate that editing plays an integral role in organismal behavior and viability.

Some evidence suggests that environmental factors, specifically temperature, affect editing of select transcripts [15-17], and that RNA structure may regulate this relationship [18,19]. For example, Garrett and Rosenthal showed that editing in an octopus delayed rectifier potassium channel transcript correlates with ambient water temperature for different species, and even suggest that editing differences contribute to octopus adaptation [20]. Additionally, auto-editing of the *Drosophila adar* (*dadar*) transcript decreases as temperature increases. Given that the ratio of edited to unedited dADAR isoforms fine-tunes the global ‘editosome’ [21] as well as complex organismal behavior, these data point toward an intriguing role of thermal control in global RNA editing [9].

However, little research has delved into the widespread effect of temperature on editing, especially in a genetically tractable organism. Because ADARs recognize duplex RNA structures, the information directing editing is carried in RNA secondary and tertiary structures, and changes in editing should partially reflect thermal alteration in RNA shapes [8]. To observe temperature effects on RNA editing we used *Drosophila*, a poikilotherm with robust RNA editing machinery and a well-characterized editosome.

Although poikilotherms experience environmental temperature undiminished, they have evolved physiological processes that allow them to thrive within the natural temperature ranges of their habitats [22]. It is possible that differential RNA editing is one process that allows poikilothermic animals such as *Drosophila* to rapidly adapt to varying environmental temperatures. Approximately 28% of ADAR-mediated RNA modifications occur in coding regions in the *Drosophila* transcriptome [23]. More specifically, A-to-I RNA editing in coding regions leads mostly to non-synonymous amino acid substitutions within proteins involved in neurotransmission [24,25]. As nervous system function is highly sensitive to temperature fluctuations [26], this suggests that editing could provide a mechanism of rapid temperature responsiveness within the nervous system.

The above observations and hypotheses led us to undertake a comprehensive survey of 54 *Drosophila* editing sites across a 20°C biologically relevant temperature range [27]. We created a high throughput batch-processing method of editing analysis to test the thermo-sensitivity of editing, which revealed diverse patterns of temperature response between different editing sites. Part of the overall pattern seen was due to altered dADAR levels and largely consistent with observations from parallel heat-shock studies [17]. Certain alterations in editing due to elevated temperature are reversible, although individual editing sites respond differently. To test the contribution of RNA structure to temperature-sensitive editing changes, we used genetically engineered *Drosophila* strains in which stabilizing and destabilizing structural mutations have been introduced into the endogenous locus of a natural editing substrate [8], revealing altered temperature sensitivity of editing due to engineered RNA structural changes. Our results suggest that RNA editing is acutely sensitive to temperature, and that this response is partially affected by the thermo-sensitive secondary and tertiary RNA structures that direct editing.

## Results

### Widespread RNA editing decreases at elevated temperature

**Figure 1:**
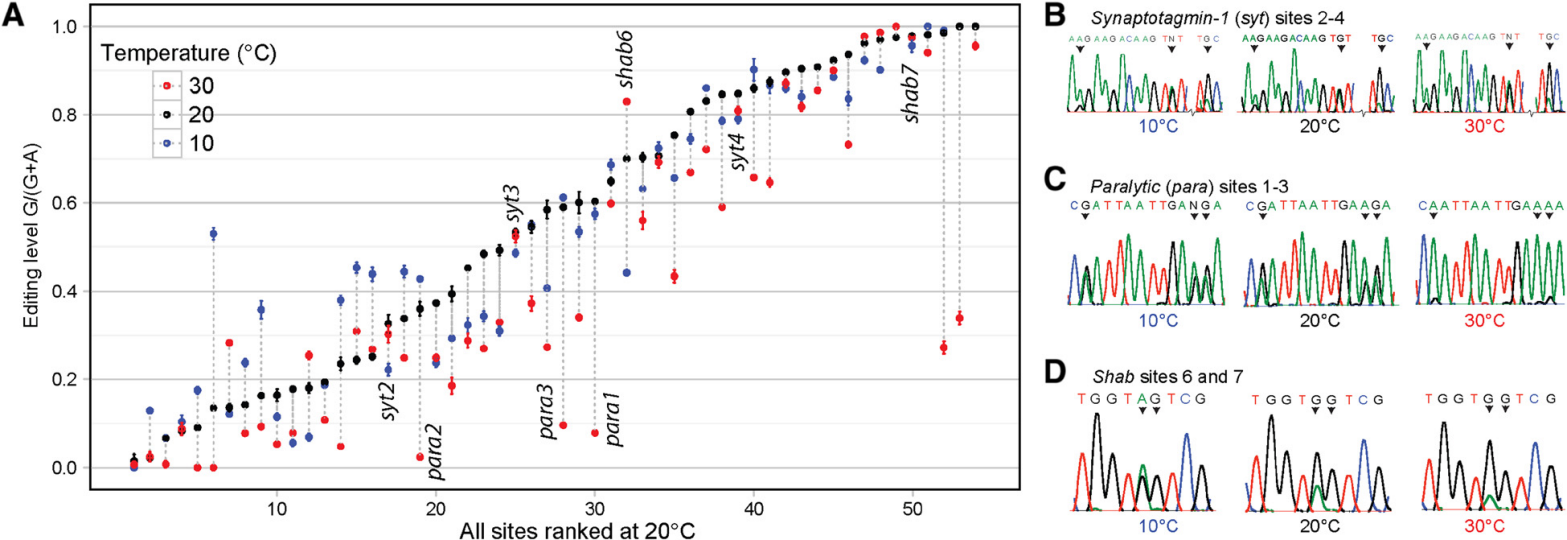
**Overall editing responses to temperature. (A)** The temperature response of fifty-four editing sites in 14 transcripts. Editing (total guanosine trace to (total adenosine + guanosine traces)) at 10°C (blue), 20°C (black) and 30°C (red) is presented for each site. All sites are ranked by editing level at 20°C. Bars represent standard error. Notable sites are labeled by gene abbreviation and adenosine site as annotated in Savva *et al*. [9]. **(B)** Editing of *synaptotagmin-1* sites 2 to 4 is largely insensitive to temperature. **(C)** Editing of sites 1 to 3 in the *paralytic* transcript decreases at 30°C. **(D)** Editing of *shab* site 6 increases with increasing temperature, while site 7, edited to 100%, is temperature-insensitive. All editing sites are indicated within chromatograms by black carrots above Sanger traces.

To investigate the effect of temperature on editing, we limited our scope to wild type Canton-S *Drosophila melanogaster*, in which the editosome has been well characterized [9]. Instead of by-hand data processing, as we have done previously [8,9,28], we created a batch processing script to remove bias, eliminate human error and increase feasibility of such a large-scale endeavor. This program does not improve upon the by-hand method, but is able to quickly process prepared files en masse using the same strategy as the by-hand method. We investigated 54 editing sites in fourteen different transcripts (see Additional file 1: Figure S1), representing the sites most highly conserved and best characterized of the over 3,000 adenosines known to comprise the *Drosophila* editosome [29]. The results from each individual site are compiled in Figures 1A, Additional file 2: Figure S2 and Additional file 3: Table S1.

We grew engineered *D. melanogaster* at 25°C before transferring newly-ecclosed male animals to equivalent incubators held at 10°C, 20°C and 30°C. We used only males to control for possible sex-specific differences. *Drosophila* eggs are most viable at 20°C [22]; therefore, the 20°C temperature range chosen is biologically relevant. After 72 hours of temperature acclimation, the animals were snap frozen at −80°C. We then performed RNA editing analysis using previously published methods [8].

Thermo-sensitive editing at individual sites occupies a range of possible patterns. For example, editing at sites 2 to 4 in the calcium sensor *synaptotagmin-1* transcript is universally insensitive to temperature (Figure 1B). However, editing of *paralytic* at sites 1 to 3 decreases significantly at 30°C (Figure 1C). Contrary to the overall trend (Figure S2A), editing of site 6 in the potassium channel *shab* transcript is potentiated by temperature, significantly increasing in editing level over the temperature range (Figure 1D). Notably, *shab* site 7, located adjacent to site 6, is edited at nearly 100%, and is temperature-insensitive within the 20°C range studied (Figure 1D).

As there are several examples of multiple editing sites within a single transcript that respond differently to temperature (see Additional file 4: Figure S3), it is unlikely that the observed changes in editing are due to fluctuating transcript levels. Thus, while the general trend is for many sites to decrease editing level at 30°C, there are notable examples that are temperature invariant, as well as those whose temperature profile is counter to the overall trend.

### Thermo-sensitive editing patterns are largely conserved

**Figure 2:**
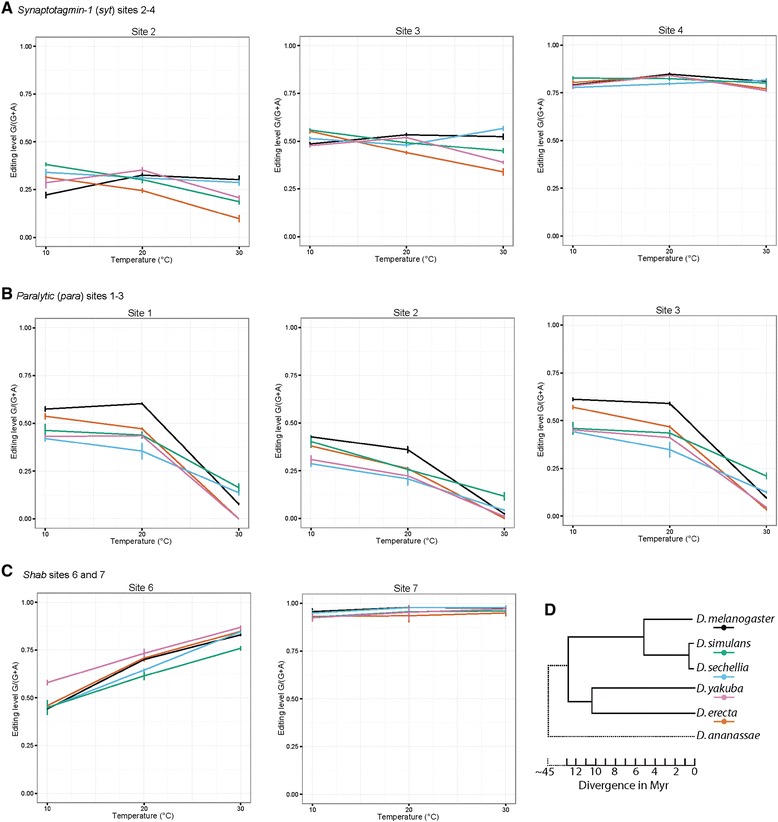
**Conservation of editing responses across** ***Drosophilidae*** **. (A)** Editing at sites 2 to 4 in the *synaptotagmin-1* transcript. **(B)** Editing at sites 1 to 3 in the *paralytic* transcript. **(C)** Editing at sites 6 and 7 in the *shab* transcript. The slopes of the species-specific editing response curves, rather than the absolute editing, were statistically compared to those of *D. melanogaster* (black) for each site. *P* <0.0001: **, *P* <0.05: *. Bars in **A** through **C** represent standard error. **(D)** Phylogeny of the five *Drosophilidae* species used in this study. *D. ananassae* represents an outgroup species. Colors are as in **A** through **C**. Branch lengths represent evolutionary time, based on Tamura *et al*. [30].

In order to determine whether the observed temperature-dependent editing responses are conserved in other species of *Drosophila*, we studied editing of select transcripts in five closely related *Drosophilidae* species (Figure 2, Additional file 5: Figure S4, Additional file 6: Table S2), representing over 10 million years of evolutionary divergence (Figure 2D) [30]. We observed that the thermo-responsive editing patterns at sites at which editing is insensitive to temperature, such as sites 2 to 4 in the *synaptotagmin-1* transcript (Figure 1B) and site 7 in the *shab* transcript (Figure 1D), are also highly conserved between the species investigated (Figure 2A and C).

The quite diverse thermo-responsive editing patterns at *paralytic* sites 1 to 3 (Figure 2B), *shab* site 6 (Figure 2C), sites 1 to 3 in the *complexin* SNARE-binding protein transcript (see Additional file 5: Figure S4A), and *dadar* auto-editing (see Additional file 5: Figure S4B) are generally highly conserved, although absolute editing varies slightly and slope also varies significantly between species (see Additional file 6: Table S2). However, editing at the single site in *uncoordinated-13*, which encodes a protein involved in calmodulin binding in the larval central nervous system, is not conserved, either in absolute levels or in response to temperature (see Additional file 5: Figure S4C, Additional file 6: Table S2).

It is possible that the slight but significant changes in thermo-responsiveness of editing between species are a result of altered RNA structures, perhaps due to single nucleotide changes or polymorphisms within the genes of the *Drosophila* species studied here. As most structures that direct editing are formed between exonic and intronic sequences, changes in primary sequence often occur in intronic *cis* elements, leading to alterations in RNA structure and a corresponding change in editing. However, it is notable that editing at both *dadar* [31] (see Additional file 5: Figure S4B) and *unc-13* (RA Reenan, personal observation, Additional file 5: Figure S4C) is directed by an entirely exonic secondary structure, whereas *synaptotagmin-1* [7] and *paralytic* [8] are sites directed by structures comprised of paired exon and intron sequences. One would, therefore, expect the structures of *dadar* and *unc-13* to be under higher sequence—and, therefore, structural—conservation, leading to highly similar editing at these sites across *Drosophilidae*. While auto-editing of the *dadar* transcript satisfies this prediction, editing in *unc-13* is highly variable (see Additional file 5: Figure S4B and C), suggesting that either the *unc-13* RNA structure or the specificity of editing machinery has evolved in the species studied, leading to the observed variability.

Our observations suggest that, in general, patterns of editing responsiveness to temperature are highly conserved and gene-specific in nature. The fact that insensitivity to temperature, temperature-sensitivity and temperature-potentiated site patterns are conserved is significant, and further suggests that while RNA structures that direct editing at certain sites are highly thermodynamically stable, others may be inherently more temperature labile, allowing editing levels to track with temperature based upon an as yet unknown interaction between temperature and RNA structure.

### ADAR protein expression level is temperature-sensitive

Changes in editing levels may manifest due to effects at the RNA level (RNA folding, interactions with transcription/splicing machinery, negative influence of RNA chaperones) and/or directly via changes in dADAR protein concentration. We assayed dADAR protein in extracts from the heads of male flies reared at the experimental temperatures, as described above. We used engineered animals in which Jepson *et al*. tagged the endogenous *dadar* gene with an HA (hemagglutinin) epitope sequence using homologous recombination (HR) [32]. The appropriate control for mutations generated via HR is an allele in which no targeted mutations have been introduced through the engineering process, but which contains a loxP sequence, the only remnant from the HR procedure, within an intron [33]. The intermediate allele generated during the HR process contains a 5.5 kb *mini-white* gene, inserted within the same intron in the *dadar* locus, which produces a viable dADAR hypomorph with approximately 20% of the protein found in the loxP ADAR+ control [32].

**Figure 3:**
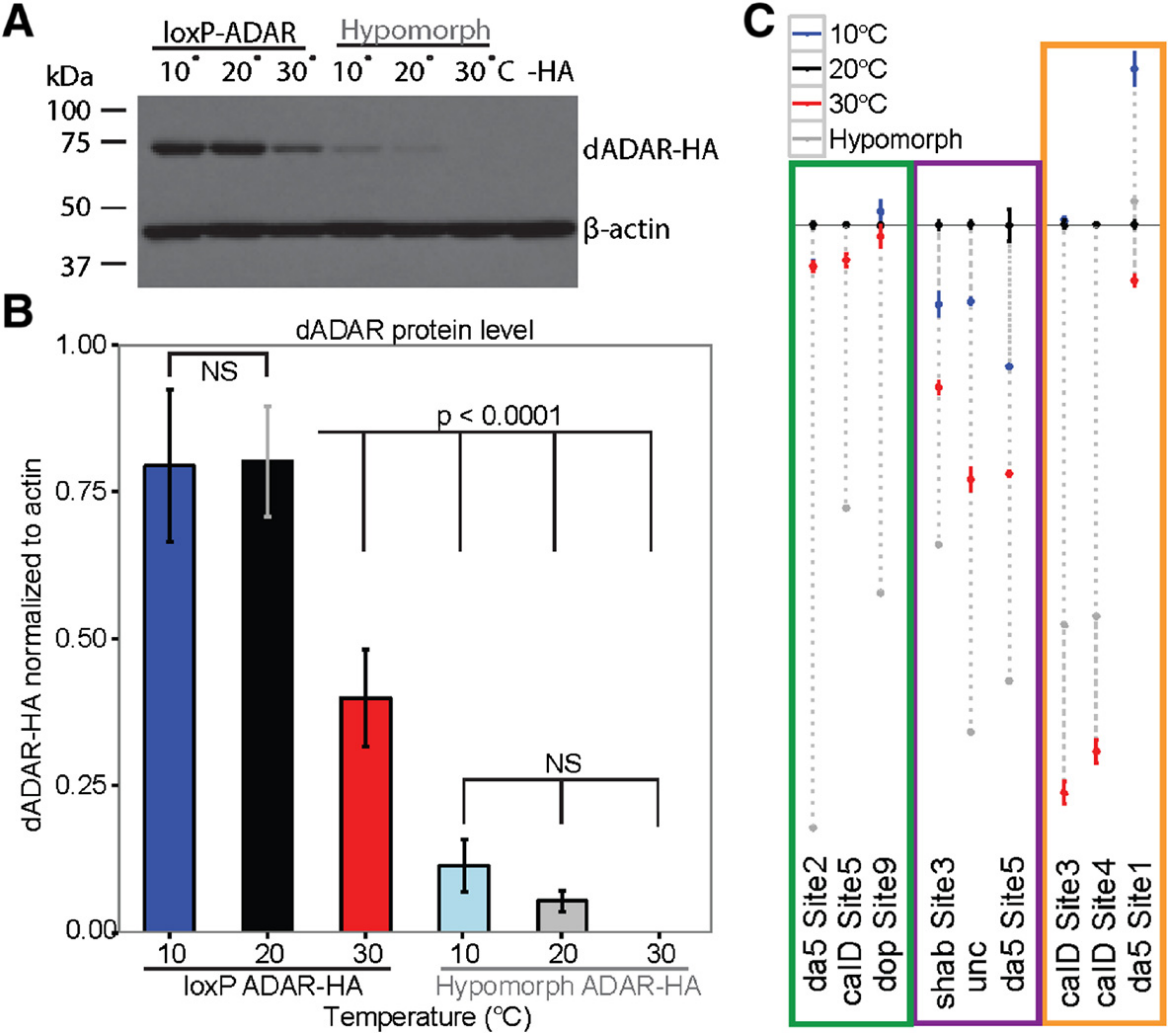
**ADAR protein level is sensitive to temperature. (A)** Western blot analysis of the HA-tagged dADAR protein, from the loxP control allele as well as the *dadar* hypomorphic allele, both generated through homologous recombination [32,33]. β-actin is presented as a loading control. Wild type dADAR, which lacks the HA tag is presented as a negative control (−HA). **(B)** Quantification of western blot analysis. dADAR-HA signal is normalized to β-actin signal from each lane. Bars represent standard deviation. **(C)** Relative editing at sites that respond to dADAR dosage and/or to temperature. Editing at 20°C (black) is aligned along the black line. Green sites are unresponsive to temperature, but sensitive in the hypomorph (gray), suggesting editing is more dependent on RNA structure than on dADAR level. Purple sites are sensitive to both temperature and dADAR levels, while orange sites are more responsive to temperature than to dADAR level, suggesting at these sites that an additional factor, such as RNA structure, is partially responsible for the response of editing to temperature.

*Drosophila* ADAR protein levels are stable between 10°C and 20°C, but decrease significantly at 30°C (Figure 3A-B, Additional file 7: Figure S5), generating a hypomorphic state. However, the amount of dADAR protein produced from a control loxP allele at 30°C is still significantly greater than that produced from the hypomorphic allele at any temperature. Moreover, the *dadar* hypomorphic allele is also sensitive to temperature, appears to experience reductions of protein levels between 10°C and 20°C and to be nearly undetectable at 30°C (Figure 3A-B). Although dADAR is completely undetectable in western blot analysis from engineered hypomorphic animals at 30°C, we observed no behavioral or phenotypic consequences. This is surprising given that dADAR null *Drosophila* display striking behavioral defects in motor control [13]. Although dADAR levels decrease when animals are kept at 30°C, editing at individual sites responds differently and independently to changes in temperature (Figure 1A), suggesting that dADAR concentration accounts for only part of the temperature responsiveness of editing sites.

Temperature-dependent editing patterns in loxP control and *dadar* hypomorphic animals are different and diverse even within a transcript (see Additional file 8: Figure S6), suggesting that dADAR levels are not fully responsible for temperature responsiveness. Finally, when editing in the hypomorph is compared to editing at different temperatures (see Additional file 9: Figure S7), it is clear that the dADAR level accounts for some, but not all, of the observed temperature dependent editing patterns (R = 0.48). Previously, we defined editing sites as ‘low-efficiency’ or ‘high-efficiency’, depending on their responsiveness to the hypomorphic *dadar* allele. Low-efficiency sites are sensitive to decreased dADAR concentrations, while high-efficiency sites are largely insensitive and are edited at a fairly constant level regardless of dADAR concentration [32]. In the present study, we manipulate the environment (30°C) to mimic the genetic hypomorph. However, the pattern of low-efficiency and high-efficiency sites is different between environmental (30°C) and genetic (hypomorph) lowered dADAR states (Figure 3 and Additional file 9: Figure S7), suggesting that the dADAR concentration is not the only factor driving editing levels and another temperature-sensitive factor, such as RNA folding, also directs editing.

### Thermo-sensitive editing response is partially reversible

**Figure 4:**
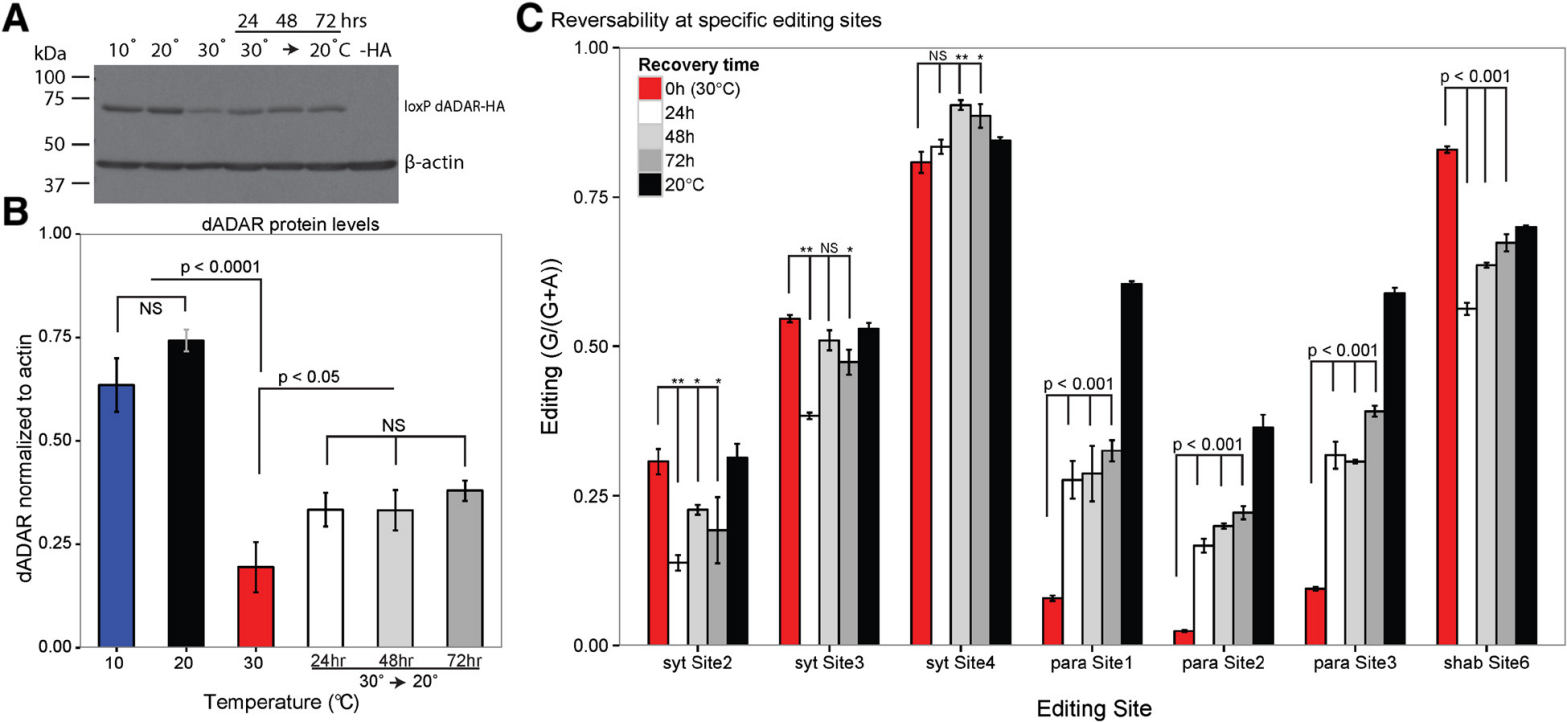
**Reversibility of temperature-induced dADAR and editing levels. (A)** Western blot analysis of the HA-tagged dADAR after three days at 10°C, 20°C and 30°C and after being shifted from 30°C to 20°C for 24, 48, or 72 hours. β-actin is presented as a loading control. Wild type dADAR, which lacks the HA tag is presented as a negative control (−HA). **(B)** Quantification of western blot analysis. dADAR-HA signal is normalized to the β-actin signal from each lane. Bars represent standard deviation. **(C)** Editing at specific sites after temperature shift. Editing after 72 hours at 30°C is depicted in red. After animals were held at 30°C for 72 hours they were shifted to 20°C for 24 (white), 48 (gray) and 72 (dark gray) hours. Animals held at 20°C for 72 hours but not shifted are depicted in black. Editing of *synaptotagmin-1* sites 2 to 4 is unresponsive to temperature (Figure 1B) yet editing at sites 2 and 3 significantly decreases after 24 hours at 20°C. These sites recover to near-20°C levels after 48 and 72 hours. Editing of *paralytic* sites 1 to 3 begins to recover from 30°C levels after just 24 hours. Editing at *shab* site 6 increases at elevated temperature (Figure 1C) and after 72 hours recovers to near-20°C levels. *Shab* site 7, edited to 100%, is unresponsive to temperature (Figure 1D) and recovery. Bars represent standard error. *P* <0.0001: **, *P* < .05: *, not significantly different: NS.

Although dADAR concentration decreases at 30°C compared to cooler temperatures (Figure 3), protein levels begin to recover after the animals are shifted to 20°C for just 24 hours, but do not fully recover even after 72 hours (Figures 4A and B, Additional file 10: Figure S8). Editing at specific sites displays varying degrees of recovery, most of which are statistically different from editing at 30°C and in the direction of editing at 20°C (Figure 4C). Editing at sites 2 to 4 of the *synaptotagmin-1* transcript is largely resistant to temperature (Figures 1B, 4C), while editing of the *paralytic* transcript at sites 1 to 3 decreases significantly at 30°C (Figure 1C); all three sites recover slowly (Figure 4C) toward 20°C levels. Editing at *shab* site 6, which increases substantially at elevated temperatures (Figure 1D), actually appears to overshoot the 20°C level and is decreased to the 10°C level after 24 hours, and stabilizes to the 20°C level after 72 hours (Figure 4C). This suggests that editing of certain sites is driven more by dADAR level, and that these sites take longer to recover from elevated temperature, while editing at other sites is driven by another factor, such as the more rapid response of RNA structure to temperature.

### Edited dADAR isoforms are not differentially sensitive to temperature

The dADAR protein auto-edits its own transcript, causing an amino acid substitution at position 458 in the deaminase domain. Unedited *dadar* transcripts produce a peptide encoding a serine at position 458 (dADAR^S^), while edited transcripts produce the glycine isoform (dADAR^G^). We previously reported that the dADAR^S^ and dADAR^G^ isoforms target the same editing sites, but edit them to slightly different levels, with the dADAR^G^ isoform displaying less activity *in vivo* [9]. Auto-editing is stable between 10°C and 20°C at about 55%, although *dadar* editing decreases to 37% at 30°C (see Additional file 2: Figure S2B, Additional file 3: Table S1), increasing the proportion of the unedited dADAR^S^ isoform. We, therefore, suspected that our observed overall temperature-dependent editing patterns (Figure 1A) might be a result of differential stability of the dADAR^S^ and dADAR^G^ isoforms.

**Figure 5:**
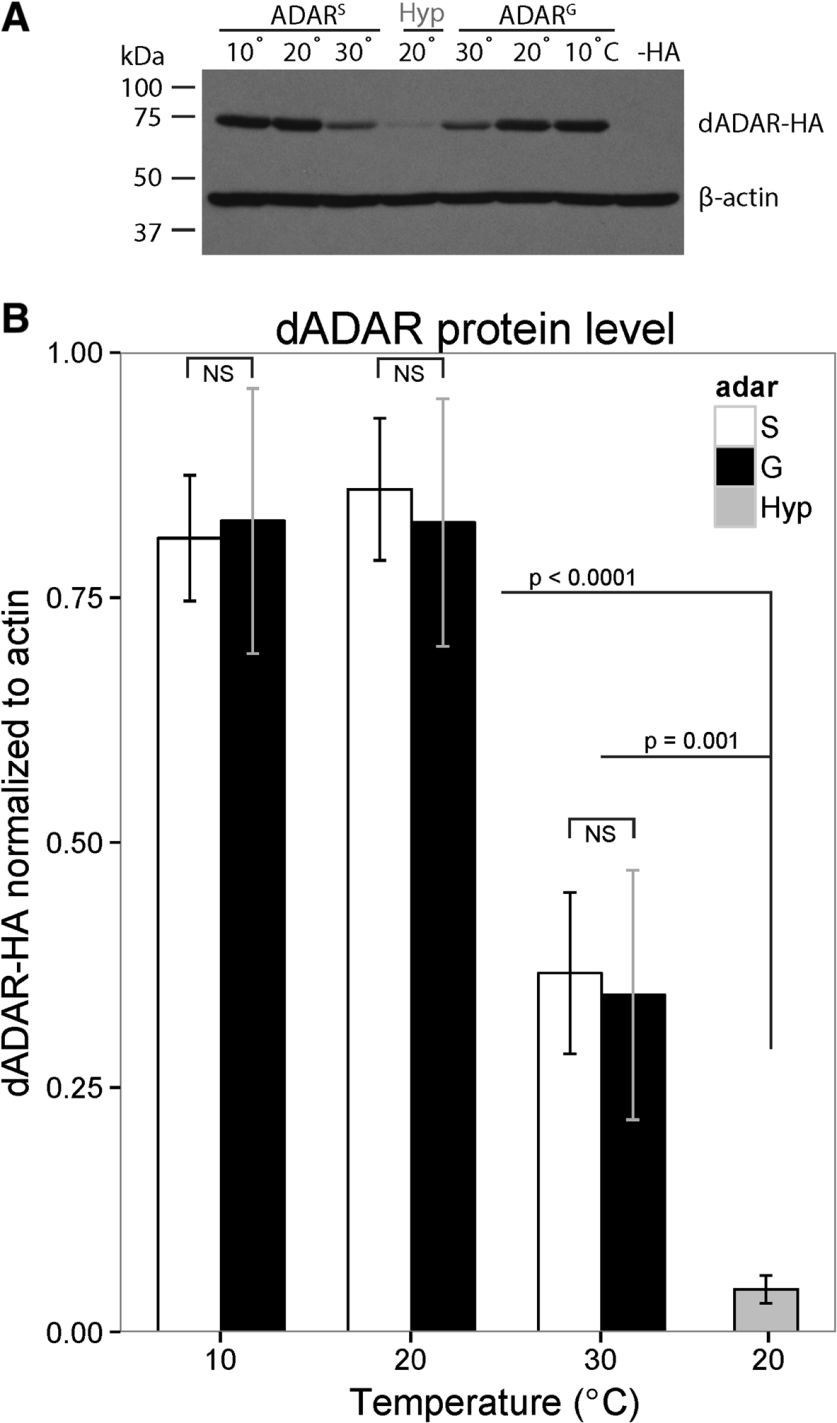
**Relative temperature stability of dADAR isoforms. (A)** Western blot analysis of HA-tagged hardwired S and G dADAR isoforms [9]. The hypomorphic allele at 20°C is presented for comparison. β-actin is presented as a loading control. Wild type dADAR, which lacks the HA tag is presented as a negative control (−HA). **(B)** Quantification of western blot analysis. dADAR-HA signal from S (white) and G (black) alleles is normalized to the β-actin signal from each lane. Bars represent standard deviation.

We previously used HR to generate engineered animals in which the dADAR^S^ and dADAR^G^ isoforms were permanently hardwired into the HA-tagged *dadar* locus. Although behavior was altered in both the dADAR^S^ and dADAR^G^ lines [9], the isoforms do not have significant differential temperature stability (Figure 5, Additional file 11: Figure S9) compared to each other and to wild type mixed isoform dADAR (Figure 3). Therefore, it is unlikely that differential temperature stability of the dADAR^S^ and dADAR^G^ isoforms is responsible for the overall changes in RNA editing apparent in Figure 1A.

### Structural RNA mutations affect temperature-dependent patterns

If RNA structures directing editing are partially responsible for the thermo-sensitivity of editing at specific sites, we reasoned that as temperature changes, the specific structures required for RNA editing may be more or less common leading to altered editing levels.

**Figure 6:**
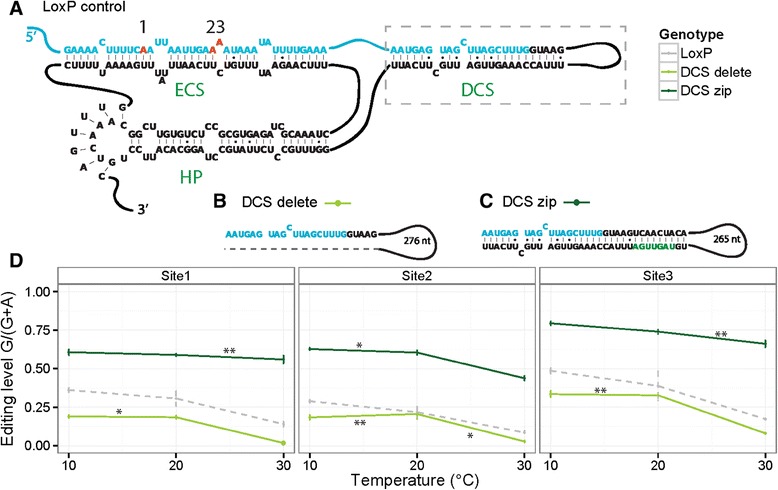
**Effect of RNA structural mutations on temperature-sensitivity. (A)** *Paralytic* editing sites 1 to 3 (red), within an exon (blue), are encompassed within a complex tertiary structure involving three intronic (black) sequences: the editing site complementary sequence (ECS), the donor site complementary sequence (DCS) and a hairpin (HP), the loop of which forms a tertiary psueoknot with a docking site 3’ to the ECS. The DCS region is boxed for comparison with the knock in structural mutations. **(B)** In the ‘DCS delete’ mutation, the DCS region of the intron is excised (gray dotted line), resulting in a loss of secondary structure. **(C)** In the ‘DCS zip’ mutation, the DCS secondary structure is extended by nine base pairs, due to the insertion of seven nucleotides (green) within the intronic sequence. **(D)** Because these mutations overall decrease (DCS delete) or increase (DCS zip) editing at all three sites [8], the slopes of the editing response curves, rather than the absolute editing, was compared to loxP. The DCS delete (light green) and DCS zip mutation (dark green) show a different temperature-sensitive response pattern than that of the loxP control (gray). *P* <0.0001: **, *P* <0.05: *.

We tested the editing thermo-sensitivity profile of knock-in RNA structural mutations in the *paralytic* voltage-gated sodium channel transcript, engineered into the endogenous locus using HR [8,33]. The RNA structure directing editing at *paralytic* sites 1 to 3 is a complex tertiary pseudoknot (Figure 6A). The intronic editing site complementary sequence (ECS) is required for editing at all three sites, while the ‘donor site complementary sequence’ (DCS), which sequesters the splice donor in secondary structure, titrates the level of editing at all three sites. Finally, the tertiary pseudoknot interaction, formed between an intronic hairpin loop and a ‘docking site’ 3′ to the ECS, selectively directs editing at site 1 [8]. We decided to use mutant lines, in which the structure of the *paralytic* transcript is altered in multiple directions, to assay the effect of temperature via RNA structure on editing levels because these sites are among the most temperature sensitive in our study (Figure 1A). We have previously published a series of mutations, engineered via HR into the endogenous *paralytic* locus. These mutations, which are described below, alter editing at *paralytic* sites 1 to 3 by perturbing the local and long-range primary and secondary RNA structure around the edited adenosines [8]. As with other instances of HR, the appropriate control for HR-engineered mutations is a loxP control allele.

The ‘DCS delete’ mutation, which is predicted to decrease secondary structure (Figure 6B), decreases editing at all three *paralytic* editing sites, while the ‘DCS zip’ mutation (Figure 6C), which increases secondary structure, is known to increase editing at all three sites [8]. The thermo-responsive editing profile of these structural mutations appears to be a product of both temperature and structure. The ‘DCS delete’ mutation displays decreased editing at all three sites compared to the loxP control, as expected, yet the thermo-responsive editing pattern, measured as slope, is significantly different at all three sites than that seen in the loxP control (Figure 6D, Additional file 12: Table S3). Similarly, the ‘DCS zip’ mutation shows decreased thermo-responsive editing patterns at all three sites compared to the loxP control (Figure 6D), in addition to increasing editing in all conditions, as expected [8]. The altered thermo-sensitivity of these mutations, defined here as slope, is very subtle, yet is still significant (see Additional file 12: Table S3).

We also tested the thermo-responsiveness of structural mutations in the *paralytic* tertiary pseudoknot structure (see Additional file 12: Table S3, Additional file 13: Figure S10 A-D). The individual pseudoknot mutations, ‘Loop > α’ and ‘Dock > α’, both disrupt the tertiary RNA interaction, and selectively abolish editing at site 1, while preserving editing at sites 2 and 3. The double mutation, ‘Loop/Dock > α/α’, combines these mutations and is predicted to restore the tertiary pseudoknot structure, rescues editing at site 1 and increases editing at all three sites [8]. These mutations reveal temperature sensitivity patterns (slope) distinct from that seen in the loxP control (see Additional file 12: Table S3, Additional file 13: Figure S10E), suggesting that engineered RNA structures are affected differently by temperature, which manifests in the thermo-sensitive editing response of *paralytic* editing sites.

## Discussion

The effects of acute environmental changes on the process of RNA editing have not previously been assessed in great detail. Here, we demonstrate the effect of temperature on RNA editing patterns using multiple known target RNAs. Our analysis reveals that editing generally decreases at 30°C, but that there are exceptions to this trend and that the temperature profile of each editing site is distinct (Figure 1A). As *D. melanogaster* exhibit fairly constant viability and fertility between approximately 12°C and 30°C [34], we suggest that the changes we observe are normal and represent an adaptive cellular response rather than a breakdown in editing efficiency. Indeed, the potentiation of certain editing sites by elevated temperature, in opposition to the overall trend, argues for a complex underlying hierarchy of responses to temperature (Figure 1D, Additional file 2: Figure S2B).

Congruent with these data is our previous observation that certain sites are more sensitive to dADAR auto-editing and concentration of dADAR protein [32]. In addition, all known editing sites have varying degrees of duplex RNA structure, although other than double-strandedness, no common sequence or structural property has been identified. This suggests that editing sites may behave differently in response to temperature because variable RNA structures act as mechanistically distinct ‘thermometers’, depending on the thermostability of the final RNA structure that directs editing at that particular adenosine, or the thermal influence on the folding pathway of that structure.

This hypothesis is supported by the observation that neighboring sites within a transcript, which are predicted to be involved in the same governing RNA structure, often show similar temperature profiles (Figure 1B-C). However, there are other transcripts (Figure 1D, Additional file 4: Figure S3) in which adjacent sites behave very differently in response to temperature. This could indicate that these sites are ‘more functionally important’ than labile sites. Indeed, with a few noted exceptions, the top 25% of sites ranked by editing level in Figure 1A are largely temperature insensitive.

We suggest that sites that are edited close to 100% are edited at this level regardless of temperature, perhaps because these structures are the most thermodynamically stable, and possible alternative structures are much less likely to form within the investigated temperature range. In support, Tian *et al*. observed that while editing of a wild type Gabra3 I/M site is insensitive to temperature, synthetic constructs containing structural mutations are temperature-sensitive. This work suggests that thermodynamically less stable structures carry an intrinsic temperature sensitivity, adding that there must be an evolutionary advantage to temperature-insensitivity at this particular site [18].

The five *Drosophilidae* species studied here encompass over 10 million years of divergent evolution (Figure 2D) and represent diverse geographical (and temperature) ranges. While *D. melanogaster* and *D. simulans* are now cosmopolitan species, *D. yakuba* is found in savannah climes [35], *D. erecta* is native to west central Africa [36], and *D. sechellia* was previously confined to the Seychelles islands [37]. Our data show that, generally, editing versus temperature profiles is conserved across species (Figure 2, Additional file 5: Figure S4, and Additional file 6: Table S2). However, editing sites that are temperature-sensitive are also more variable across species, suggesting that labile RNA structures are under less selective pressure.

One mechanism through which temperature affects RNA editing is through dADAR concentration. We found that dADAR concentration is stable between 10°C and 20°C, but significantly decreases at 30°C (Figure 3), as does RNA editing for many sites (see Additional file 2: Figure S2A). However, levels of the auto-editing isoforms of the dADAR protein do not show differential sensitivity to temperature (Figure 5). The editing enzyme is likely to play an important role in adaptation to temperature; Ma *et al*. discovered that the hypnos-2 mutant allele of *dadar* resulted in a loss of editing and increased susceptibility to heat-shock [38]. It is possible that decreased dADAR expression at 30°C is adaptive outside of the direct impact on widespread or specific editing. For example, ADAR is also implicated in the RNAi pathway. ADAR is known to edit RNAi precursors and also competes with the RNAi machinery for double-stranded RNA substrates [39,40]. Further, ADARs affect gene expression through heterochromatic gene silencing [41], providing a possible evolutionary significance for the sensitivity of dADAR expression to temperature. Thus, temperature alterations can change gene expression through dADAR concentration.

To test our overall hypothesis, that the responsiveness of RNA editing to temperature is mediated in part by RNA structure, we tested several knock-in mutations in the *paralytic* locus [8]. These mutants were designed to probe and confirm secondary and tertiary RNA base pairing interactions directing the editing of three particular adenosines. In fact, these three adenosines in this unique and complex RNA structure are among the most temperature sensitive ADAR editing sites we observed (Figure 1B, Additional file 3: Table S1). Our mutations represent bidirectional changes to both secondary (DCS, Figure 6) and tertiary (pseudoknot, Additional file 13: Figure S10) structural elements.

Interestingly, in this particular transcript, editing level appears to be responsive to both structure and temperature in an additive manner (Figure 6D, Additional file 13: Figure S10E), suggesting levels of regulation at both the molecular and abiotic levels. Combining mutations designed to restore the pseudoknot structure does not restore the thermo-sensitivity of editing to loxP control levels. This is likely due to altered stability of the RNA tertiary structure: we previously showed that combining the Loop > α and Dock > α’ mutations rescues editing at *paralytic* site 1, but also results in increased editing at all three sites compared to loxP [8]. This mutational pair (α-α’) recreate a kissing loop interaction that is substantially stabilized by additional hydrogen bonds and potentially increased stacking energies, suggesting that slightly altered stability of the structure alters both absolute editing level as well as thermo-sensitivity of the whole multi-duplex structural complex encompassing the three *paralytic* sites.

The most conclusive results from these mutants suggest the stabilization of editing levels against temperature by mutations that increase editing, such as the DCS zip and Loop/Dock > α/α’ mutations. Both of these mutations increase editing at all three sites and also result in less thermo-sensitive editing profiles for *paralytic* sites (Figures 6 and Additional file 13: Figure S10). These mutations likely increase the thermo-stability of secondary and tertiary structures around these sites by adding base pairing interactions or stabilizing base pairs. However, this observation is specific to these particular sites in *paralytic,* in the most complex RNA tertiary structure yet described for a dADAR substrate, and is not necessarily representative of global trends.

RNA editing is intricately linked to splicing [6]. Splicing often occurs co-transcriptionally [42] and many of the RNA structures that direct editing involve the participation of intronic *cis* elements. ADAR must, therefore, edit before RNA structures are resolved and removed to allow transcript splicing. For example, the *paralytic* HR mutation DCS zip causes the splice donor to be sequestered in an additional secondary structure. This results in increased editing at all three sites in *paralytic*, but also results in a splicing defect and a temperature-sensitive phenotype [8]. In the present study, we chose PCR primers in exons adjacent to the editing sites of interest and did not observe any temperature-dependent alternative spliceoforms after RT-PCR (see Additional file 14: Figure S11). These data reflect splicing decisions local to the editing sites of interest, and long-range splicing may be affected. Additionally, temperature-sensitive ADAR recruitment may compete with splicing machinery, especially since the structures that direct editing are often formed between exon and downstream intron, and many editing sites are located near exon-intron boundaries [6].

**Figure 7:**
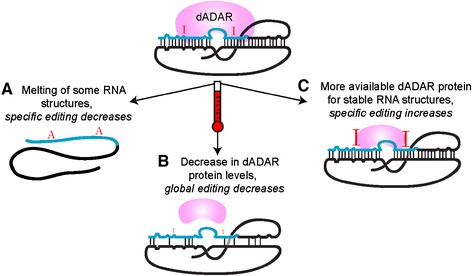
**Molecular model of the effect of temperature on RNA editing. (A)** At increased temperature, some RNA structures that direct RNA editing, formed between sequences in an exon (blue) and intron (black), melt, resulting in a decrease in editing at 30°C. **(B)** dADAR protein (pink) level decreases at elevated temperatures, leading to a decrease in editing at the RNA structures that still form at 30°C. **(C)** Because some RNA structures melt (**A**), dADAR protein, although present at lower concentrations, is free to edit remaining highly stable RNA structures, leading to an increase in editing at certain adenosines.

Based on the data presented above, we propose that temperature affects editing in at least three ways (Figure 7). First, elevated temperature melts some labile RNA structures, leading to a decrease in editing at the adenosines encompassed by the structure (Figure 7A). Second, dADAR concentration decreases at elevated temperatures, leading to a decrease in overall editing, even at some more stable RNA structures whose binding efficiency for ADAR may be less avid (Figure 7B). Finally, although dADAR concentration is decreased overall at higher temperatures, the melting of some RNA structures releases enzyme that may bind non-specifically, to edit adenosines in thermodynamically stable structures, leading to an increase in editing at certain sites (Figure 7C). In addition, it is possible that, certain cellular mechanisms, such as heat-shock chaperone proteins, may assist in the folding of some less stable RNA structures, potentiating editing at specific adenosines.

The temperature range studied here is biologically relevant to *Drosophila*, and an individual animal is likely to experience the full 20°C temperature range many times during a one to two month lifetime on many temporal scales. Therefore, poikilothermic *Drosophila* require mechanisms of rapid and reversible cellular adjustment, especially at the level of the critical neuronal proteome, considering the intrinsic temperature sensitivity of neuronal signaling. Although the present study does not address whether or not the response of editing to temperature is adaptive in *Drosophila*, editing in a number of the potassium channel transcript sites in this study is known to affect channel function.

For example, five highly edited sites in the *shab* potassium channel transcript affect the voltage-dependence and kinetics of the resulting channels [43]. Characterization of nine Shaker potassium channel isoforms resulting from editing at four sites in the *shaker* transcript revealed functional epistasis of edited residues on channel inactivation [44], and gating currents of the Shaker channel are known to be highly temperature sensitive [45]. Finally, editing of four adenosines in the *ether-a-go-go* potassium channel transcript results in altered channel activation and inactivation kinetics, and editing at a single site alters channel sensitivity to extracellular Mg^2+^ concentrations [28]. Based on these data, it seems likely that flies living at a different temperature for more than a day or two would be expressing channels altered in their editing profile, and altered in their neuronal signaling properties. We envision that such environmental modulation of channel properties plays a significant and possibly adaptive role in tuning channel kinetics in response to temperature.

Additional intriguing evidence for the role of editing in temperature adaptation exists in the literature. The timing of *Drosophila* embryogenesis is affected by temperature [46], and dADAR is expressed in the developing embryo in multiple tissues, including the developing nervous system [47]. Although editing is not known to occur early in development at substantial levels, dADAR may play an additional cellular role in temperature-dependent developmental timing. For example, the relative timing of developmental events scales across temperatures and is constant between many species [48].

Garrett and Rosenthal have suggested that, due to its unique molecular properties, RNA editing may act as a mechanism for cold adaptation and acclimation because the conversion of an adenosine into an inosine often results in the coding shift to a smaller amino acid side chain [49]. A prevailing hypothesis in the field suggests that enzyme isoforms with increased flexibility around an active site require a lower enthalpy of the rate-limiting step of catalysis, leading to a decrease in cold sensitivity. Because editing often results in amino acid substitutions, changes at the RNA level via editing could affect temperature sensitivity at the protein level.

Our survey of 54 editing sites represented a broad sample of the amino acid changes possible through RNA editing [6]; the current analysis includes examples of all 17 amino acids created or destroyed via editing. However, we did not see any overall evidence of edited amino acid directionality due to temperature (see Additional file 15: Figure S12). While our data do not support the idea that editing is an adaptive response to temperature by increasing active site lability, as suggested by Garrett and Rosenthal, they also do not preclude this possibility. Further studies in diverse organisms and ADAR targets are necessary.

## Conclusions

RNA structures possess the ability to respond to biotic signals, including molecular ligands, and abiotic signals, such as temperature, regulating diverse cellular tasks such as transcriptional regulation and protein synthesis. These structures are inexorably linked to RNA processing events, including RNA editing. Here, we present a survey of the response of RNA editing to temperature alterations in *Drosophila*. Our results indicate multiple molecular mechanisms of temperature response, including RNA structural stability and dADAR concentration. We found that individual editing sites behave very differently in response to a range of temperatures, suggesting that editing of some adenosines is predominantly influenced by RNA structure, while others are governed by additive mechanisms including enzyme concentration. These data present the first evidence supporting the existence of RNA editing ‘thermometers’, which directly regulate editing in response to environmental temperature. It will be interesting to determine the effects of temperature and other environmental cues on the other functions of ADAR as a regulator of heterochromatic gene silencing [41].

## Methods

### *Drosophila* stocks

We raised wild type (Canton-S) *Drosophila* at 25°C in humidity-controlled incubators with 12-hour light/dark cycles. As age is known to affect editing [50], we collected animals daily as they ecclosed and shifted newly emerged animals to one of three temperatures: 10°C, 20°C and 30°C, using temperature, humidity and light-controlled (12-hour light/dark) incubators. From experience we know that flies can live within this temperature range for 72 hours, although they are most often subjected to natural temperatures between 20°C and 30°C. We allowed these animals to remain in the incubators for 72 hours, and then immediately froze flies from this population for long-term storage at −80°C.

Additionally, we raised and collected *D. erecta*, *D. sechellia*, *D. simulans* and *D. yakuba* in the same manner as *D. melanogaster* above. We did the same with mutant *D. melanogaster* in which the endogenous *dadar* gene had been HA-tagged using homologous recombination, as well as the dADAR hypomorph [32], the dADAR^S^ and dADAR^G^ engineered animals [9] and engineered *Drosophila* with mutations in the *paralytic* locus [8].

### RNA editing analyses

For each unique species/genotype/temperature sample, we extracted RNA from male fly heads (N = 15 to 20) using TriReagent (Molecular Research Center, Inc., Cincinnati, OH). We chose to use only males to control for sex-related editing changes. For each sample, three separately extracted RNA biological replicates were used. We amplified cDNAs via RT-PCR using random primers, performed PCR using target-specific primers (two per technical PCR replicate per RNA sample) and electrophoresed samples on agarose gels. We cleaned the PCR products using Wizard Gel and PCR Cleanup Kits (Promega, Madison, WI) and detected editing by Sanger sequencing (University of Wisconsin Biotechnology Center, Madison, WI, USA). For species-specific editing, we designed primers to regions of high sequence conservation.

### *In silico* editing quantification

We wrote a program to calculate the ratio of mixed peak areas in chromatograms generated by Sanger sequencing. This program replicates and replaces the procedure that was previously done by hand [28], generating more accurate, consistent and timely results that correlate highly with those generated by hand. For each mixed chromatogram peak, the program truncates the curves to mitigate the influence from adjacent curves. It then uses linear interpolation to complete parts of the curve missing due to graphical artifacts and linear extrapolation to reconstruct the truncated tails of the curves; higher-moment extrapolation showed little effect on the results in practice. Finally, it computes the area underneath each curve to calculate the mixed peak ratio of the curves, which is a number between 0 and 1 corresponding to the editing level G/(G + A).

### Western blot analysis

We prepared whole-head lysates from 30 male heads per 100 μl radioimmunoprecipitation assay (RIPA) buffer (150 mM NaCl, 1.0% NP-40, 0.5% sodium deoxycholate, 0.1% SDS, 50 mM Tris–HCl, pH 8.0), including 1 mM dithiothreitol (DTT), 1 mM phenylmethanesulfonylfluoride (PMSF) and protease inhibitor mixed tablet (Roche). We mixed approximately 25 μg protein per sample with 6 X sample buffer containing SDS and β-mercaptoethanol and loaded samples on 12.5% SDS-PAGE gels, which we then transferred to a 0.2 μm pore size polyvinylidene difluoride membrane (Bio-Rad). Primary antibodies included anti-actin (Millipore; MAB1501; 1:40,000), and anti-HA (Covance; MMS-101P; 1:750). The secondary antibody used was horseradish peroxidase (HRP)-conjugated goat anti-mouse (Abcam; ab5930; 1:6,000). Both technical replicates and biological replicates were performed for each analysis and are presented as supplemental figures. We performed densitometry using ImageJ analysis software (US National Institutes of Health, Bethesda, MD, USA).

### Statistical analyses

For editing analyses, we performed one-way analyses of variance ANOVAs (α = 0.05) followed by Dunnett *post-hoc* tests in which editing at 10°C and 30°C was compared to editing at 20°C. Exact *P*-values are noted in supplemental tables (see Additional file 3: Table S1, Additional file 6: Table S2, Additional file 12: Table S3) and on graphs where possible. To compare curve shapes, a population of slopes was calculated from biological and statistical replicates. The populations of slopes between 10°C and 20°C, and 20°C and 30°C for all mutations/species were compared to the appropriate controls (*D. melanogaster* for Figures 2 and Additional file 4: Figure S3, and loxP for Figures 6 and Additional file 13: Figure S10) using one-way ANOVAs (α = 0.05) followed by Dunnett *post-hoc* tests.

## Additional files

Additional file 1: Figure S1. Sequences surrounding all editing sites. Editing sites are indicated as highlighted red adenosines. Superscripts indicate site numbers. Yellow highlighted asterisks indicate the position of an intron that has been removed in the mature transcript. Additional file 2: Figure S2. Global editing decreases at 30°C. **(A)** Editing (total guanosine trace to (total adenosine + guanosine traces)) at 10°C (blue), 20°C (black), and 30°C (red) is presented for each site. The same editing sites presented in Figure 1A are now ranked independently at each temperature. **(B)** All editing sites from Figure 1A are annotated by gene and site as in Savva *et al*. [9]. Additional file 3: Table S1. Raw data for all temperatures and editing sites. Red sites are highly edited while those in green are edited at a very low level. Standard error is presented for each site at all temperatures. Additional file 4: Figure S3. Examples of different temperature patterns in sites within the same transcripts. **(A)** The *shab* transcript contains three editing sites, which respond differently to temperature. **(B)** The *Ca-alpha1D* (ca1D) transcript is edited at five sites, each of which displays a different editing pattern in response to temperature. Additional file 5: Figure S4. Conservation of additional editing site responsiveness across *Drosophilidae.* **(A)** *Complexin* sites 1 to 3 are largely temperature unresponsive and these patterns are also conserved across all species studied. **(B)** Auto-editing of the *dadar* transcript is stable between 10°C and 20°C, but decreases at 30°C, a pattern that is mostly conserved except in *D. erecta.* **(C)** Editing at the single adenosine in the *uncoordinated-13* transcript is highly temperature sensitive and does not appear to be conserved between *Drosophilidae* species. Bars represent standard error in **A** through **C**. The slopes of the species-specific editing response curves, rather than the absolute editing, were statistically compared to those of *D. melanogaster* (black) for each site. Statistics are presented in Esm 6: Table S2. **(D)** Phylogeny of *Drosophilidae* species studied, as in Figure 2D. *D. ananassae* is presented as an outgroup. Additional file 6: Table S2. Raw data for all the editing sites surveyed across species. Red sites are highly edited while those in green are edited at a very low level. Standard error is presented for each site at all temperatures. Additional file 7: Figure S5. Raw western blot data used for quantification in Figure 3B. Western blot analysis of the HA-tagged dADAR, as well as the dADAR hypomorph, both generated through homologous recombination [31]. β-actin is presented as a loading control. Three biological and two technical replicates are shown and indicated. Additional file 8: Figure S6. Editing sites respond differently to temperature in the dADAR hypomorph. Editing is presented for six sites in the *ether-a-go-go* transcript, three sites in *synaptotagmin-1*, three sites in *paralytic*, eight sites in the *nicotinic acetylcholine receptor* α*5* transcript, five sites in *shab*, and a single site in the *uncoordinated-13* transcript. Solid lines represent the editing pattern in the loxP dADAR control, while dotted lines represent the pattern due to the hypomorphic *dadar* allele [31]. The editing pattern of some sites, for example *ether-a-go-go* site 4 and *shab* site 4, changes very little due to decreased levels of dADAR. Other sites, for example *ether-a-go-go* site 6 and *nicotinic acetylcholine receptor* α*5* sites 2 and 3, change in scale due to decreased dADAR enzyme. Still other sites, including the single site in *uncoordinated-13* and *shab* site 6, display a different temperature responsive pattern in the hypomorph compared to the control. Additional file 9: Figure S7. Editing responsiveness to temperature and dADAR level. The hypomorphic *dadar* allele results in decreased dADAR protein levels. Here, editing at specific sites in the hypomorph ([32]; gray) is overlaid with the temperature data presented in Figure 1A. A subset of sites is plotted, as not all sites from the current study were assayed in Savva *et al*. While editing at most sites decreases in the hypomorph, even compared to editing in the wild type animal at 30°C, each site responds differently to temperature changes and dADAR concentration. These data suggest that while decreasing dADAR protein may account for some decrease in editing at 30°C, there is some other factor, for example RNA structure, that is also affected by temperature and impacts editing level. Highlighted sites are reproduced in Figure 3C. Additional file 10: Figure S8. Raw western blot data used for quantification in Figure 4B. Western blot analysis of the HA-tagged dADAR and the β-actin control after temperature shift and recovery. Three biological replicates and technical replicates are presented and labeled. Additional file 11: Figure S9. Raw western blot data used for quantification in Figure 5B. Western blot analysis of the HA-tagged dADAR^S^ and dADAR^G^ isoforms of dADAR [9] across temperatures. Protein levels from the hypomorphic allele (Hyp) at 20°C are presented for comparison. Wild type dADAR, which lacks the HA tag is presented as a negative control (−HA). β-actin is presented as a loading control. Two biological replicates and two technical replicates are presented and labeled. Additional file 12: Table S3. Raw data and significance for all *paralytic* editing sites in engineered mutants. Red sites are highly edited while those in green are edited at a very low level. Standard error is presented for each site and mutation at all temperatures. The slope was calculated for each mutant from 10°C to 20°C and 20°C to 30°C and compared to that of the loxP control. *P*-values are indicated from one-way ANOVAs (α = 0.05) followed by Dunnett *post-hoc* tests. Additional file 13: Figure S10. Effect of tertiary RNA structural mutations on temperature-sensitivity. **(A)** *Paralytic* editing sites 1 to 3 (red), within an exon (blue), are encompassed within a complex tertiary structure involving three intronic (black) sequences: the editing site complementary sequence (ECS), the donor site complementary sequence (DCS) and a hairpin (HP), the loop of which forms a tertiary pseudoknot with a docking site upstream of the ECS. The pseudoknot region is boxed for comparison with structural mutations. **(B)** In the ‘Loop > α’ mutation, the loop region of the hairpin (HP) is mutated at three nucleotides (green), resulting in a loss of tertiary structure and editing at site 1. **(C)** In the ‘Dock > α’ ‘ mutation, the docking site region is mutated at three nucleotides (green), resulting in a loss of secondary structure, as well as editing at site 1. **(D)** In the ‘Loop/Dock > α/α’ ‘ mutation, the previous two mutations are combined (green) to restore the tertiary structure and site 1 editing. **(E)** Because these mutations themselves may result in an overall increase or decrease in the absolute level of editing at all three sites [8], the slopes of the editing response curves, rather than the absolute editing, were compared to that of loxP. The Loop > α (light purple) and Dock > α’ mutation (dark purple) abolish editing at site 1, as shown previously [8], and confer a different editing pattern in response to temperature on sites 2 and 3, compared to the loxP control (gray). The rescue mutation, Loop/Dock > α/α’ (blue) increases editing at all three sites, as shown previously, but also alters the temperature-sensitive response editing pattern in all three sites compared to that of the loxP control (gray). *P* <0.0001: **, *P* <0.05: *, *P* <0.0001: **, *P* <0.05: *. Additional file 14: Figure S11. Representative PCRs from cDNAs showing no change in splicing. PCR products were designed to span exon-exon boundaries surrounding the editing sites. Three replicate PCRs from animals held at each temperature from **(A)** *synaptotagmin-1*, **(B)** *paralytic* and **(C)** *shab* show no splicing change in the vicinity of the editing sites. Lane 1 in each gel shows a 100-bp ladder. This was representative of all the PCR reactions conducted in this study. Additional file 15: Figure S12. Relative proportion of amino acids from targeted codons at each temperature. The total proportion of each amino acid in all transcripts, edited and unedited, is presented for 10°C (blue), 20°C (black) and 30°C (red). The codons for 17 amino acids (and all three stop codons) contain at least one adenosine. The only amino acid not represented in our analysis is tryptophan. Editing sites that introduce stop codons were discarded for this analysis. Note that the isomers leucine and isoleucine are combined. The molecular weight of each amino acid is presented in KDa in parentheses.
