# Supplementary material for: Dynamic response of RNA editing to temperature in Drosophila
Source: BMC Biol. 2015 Jan 3;13:1. doi: 10.1186/s12915-014-0111-3 (PMC4299485; doi:10.1186/s12915-014-0111-3)
Supplement: Additional file 1: Figure S1. — Sequences surrounding all editing sites. Editing sites are indicated as highlighted red adenosines. Superscripts indicate site numbers. Yellow highlighted asterisks indicate the position of an intron that has been removed in the mature transcript. [file 12915_2014_111_MOESM1_ESM.pdf]

* = intron location
A = editing site

adar (CG12598):
CAGCACCTTGTGGGGATGCACGGATATTTA[1]GTCCTCACGAAAACGACACTGG
TGTTGATAAAC

caps (CG33653):
GACACAAGGTCTAAGCGGTAAACTTA[1]TGTCTGTGCTAGAGTCGACTTTGTC

caID (CG4894):
CAGCGGAATGTGTTATGAAAATTTTA[1]GCATATGGTTTTGTGTTACATA[2]ATGG
TGCATATCTA[3]A[4]GAAATGGATGGAATTTATTAGATTTATTAGATTTACAAT
TGTAGTTATA[5]GG*GGCGATAAGTACTGCACTCTCCC

cpx (CG32490):
AACTGAAAAATCAAATA[1]GAAACGCAAGTAA[2]A[3]TGAGCTAAAAACTCAAATA
GAGGGAAAATGTGTCATGCAGTGA

da5 (CG32975):
CTACTTCCGATGCGGTGCCATTGCTGG*GTA[1]CATATTTCAATTGCATAATGTT
TATGGTAGCTTCATCCGTTGTGTCAACGATTTTA[2]A[3]TATTAAATTATCATCATC
GAAATGC

TGACAGACTGTGCCTTATCATATTCACAATGTTCA[4]CAA[5]TATTA[6]GCCACAAT
A[7]GCTGTACTACTA[8]TCA[9]GCACCACATATTATTGTCTCGT

dop (CG18314):
AACTGCTCTGGCTGAAAA[1]TTTA[2]TCGAACCCTA[3]GTCATA[4]TGATGTCATTCGC
ATTA[5]GTCTTTGCATTCTGGGTGTCCTGGCTGCCATGGATTCTG

TGCTTAACCATTTGTTGTAAGACTA[6]AGGGCCGTTTGCAAGCAGAGCTA[8]A[9]TC
GGGTTGGACCCAGATGACTA[10]GAGTTAGATTTAGATTTTCATTCTCC

eag (CG10952):
CTGGATACCGAGAAG*GTACTAAACTA[2]TTGTCCGAAAGATATGAAGGCTGAC
TATGTGTTCATCTAAATCGCAAAGTA[3]TTTA[4]ACGAGCATCCGGCA[5]TTTCGTCT
GGCCTCGGATGGTTGTC

CTCAGACATCGACTGATTTTTCGCAA[6]GGTGGCCGATGTGAAGCGCGAAAAA

para (CG9907):
CAATTTACGACTGAAAACTTTTCA[1]ATTAATTGAA[2]A[3]ATAAATATTTTGAAAC
AGCTGTTATCACTATG

rdl (CG10537):
GTCAATTTTGGACCGATCCTCGTTTA[1]GCGTATA[2]GAAAACGACCTGGTGTAGA
AACACT

CTACATACCCTCTGGACTGATCGTTA[3]TTATATCATGGGTATCATTTTGGCTAT
GGGTATCATTTTGGCTCAATCGCA[4]ATGCAACGCCGGCGCGTGTGGCGCT

GTCGGCTACATGGCAAAACGAATTCAA[5]A[6]TGCGAAAACAAAGATTTATGGCG
AT

sbd (CG6798):
GCCCAGCGATTCGGGCGAAAAG*GTTA[1]CGTTA[2]TGTATTTCGATACTCGTGTC

shab (CG1066):
TCGTTTCAATACCGGAAA[3]CATTTTGGTGGGCGGGTATTACAATGACAACTGT
TGGCTACGGGGACATCTA[4]TCCCACAACTGCACTGGGAAAGGTTATTGGTA[5]C

CTGTGTGTTGCATATGCGGTGTTCTGGTA[6]A[7]TCGCTTTGCCTATTCCCATCATC
GTTAACAATTTTGCTGAATTTTATAAGAATCAG

stn (CG40306):
GTTTACTATGCCCGCCACGCAGGTCTCCCATA[1]CCACCGTGCGCTCGGTGAGT
GTCCAGGATTCGGATGGCGATGAACCGC

syt (CG3139):
TTGAAGAAGATGGACGTGGGCGGATTGTCTG*ATCCATATGTGAAAATTGCA
ATCATGCAAAATGGCAAACGTTTGAAAAAGAA[2]GAAGACAAGTA[3]TCAAAAA
ATGCACCCTCAACCCTTACTATAATGAGTCGTTCTCATTTGAAGTACCATTTG
AACAAATA[4]CAA*AAAATCTGTCTCGTTGTGACCGTCGTGGACTACGATCGTA
TTGGCACCTCCGAACCCATCGGCCGCTG

unc (CG2999):
ACGCAATTGACGCAGTGTTTCGATGTTGTTA[1]GCAAACTCGAGTGTCCTGATC
CAGAAATTTGGA
