# Supplementary figures and images for: Dynamic response of RNA editing to temperature in Drosophila
Source: BMC Biol. 2015 Jan 3;13:1. doi: 10.1186/s12915-014-0111-3 (PMC4299485; doi:10.1186/s12915-014-0111-3)

**A.** Global Editing as a Function of Temperature

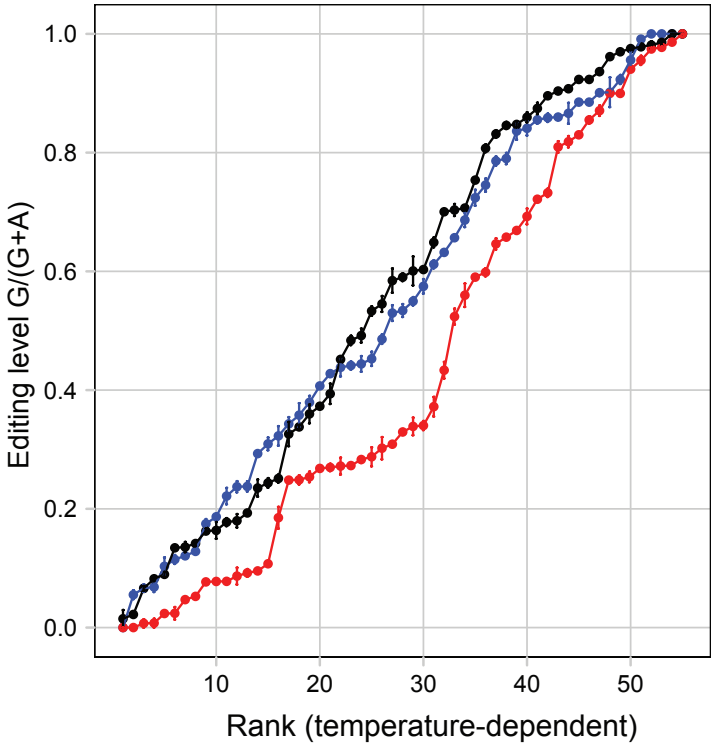

**B.** Global Editing as a Function of Temperature

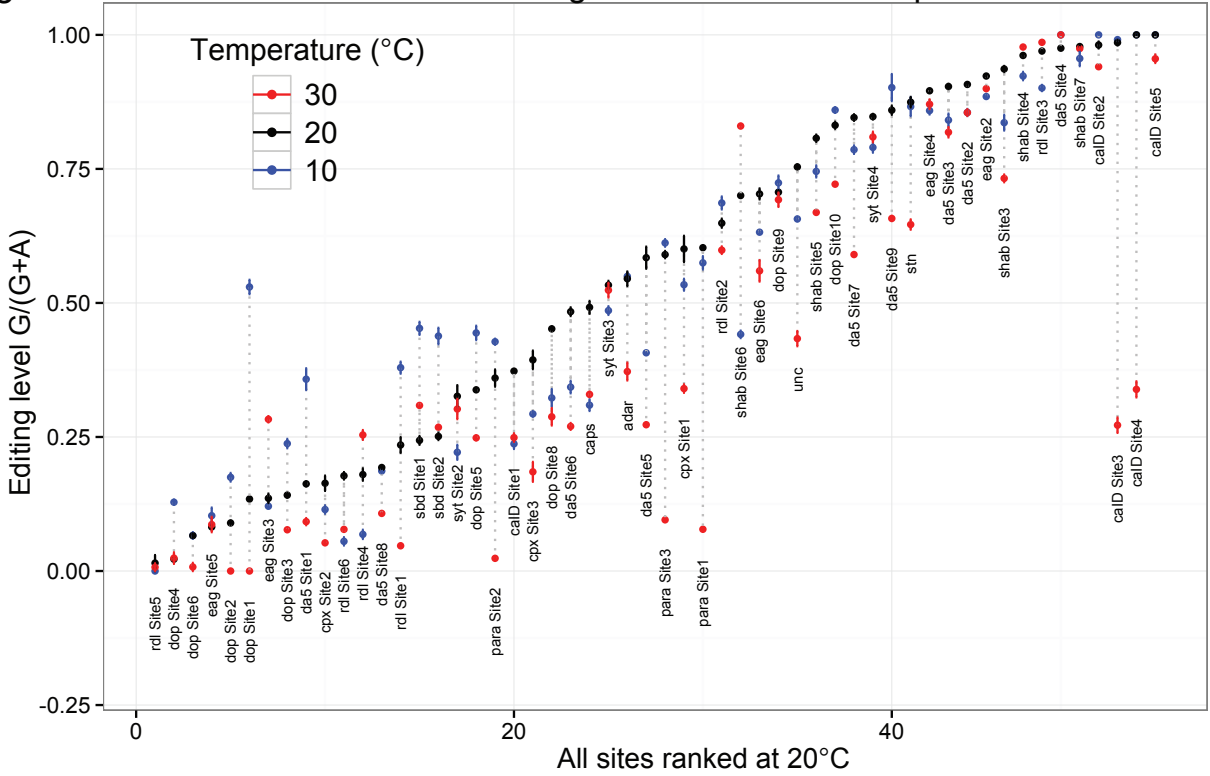

Supplement: Additional file 2: Figure S2. — Global editing decreases at 30°C. (A) Editing (total guanosine trace to (total adenosine + guanosine traces)) at 10°C (blue), 20°C (black), and 30°C (red) is presented for each site. The same editing sites presented in Figure 1A are now ranked independently at each temperature. (B) All editing sites from Figure 1A are annotated by gene and site as in Savva et al. [9]. [file 12915_2014_111_MOESM2_ESM.pdf]

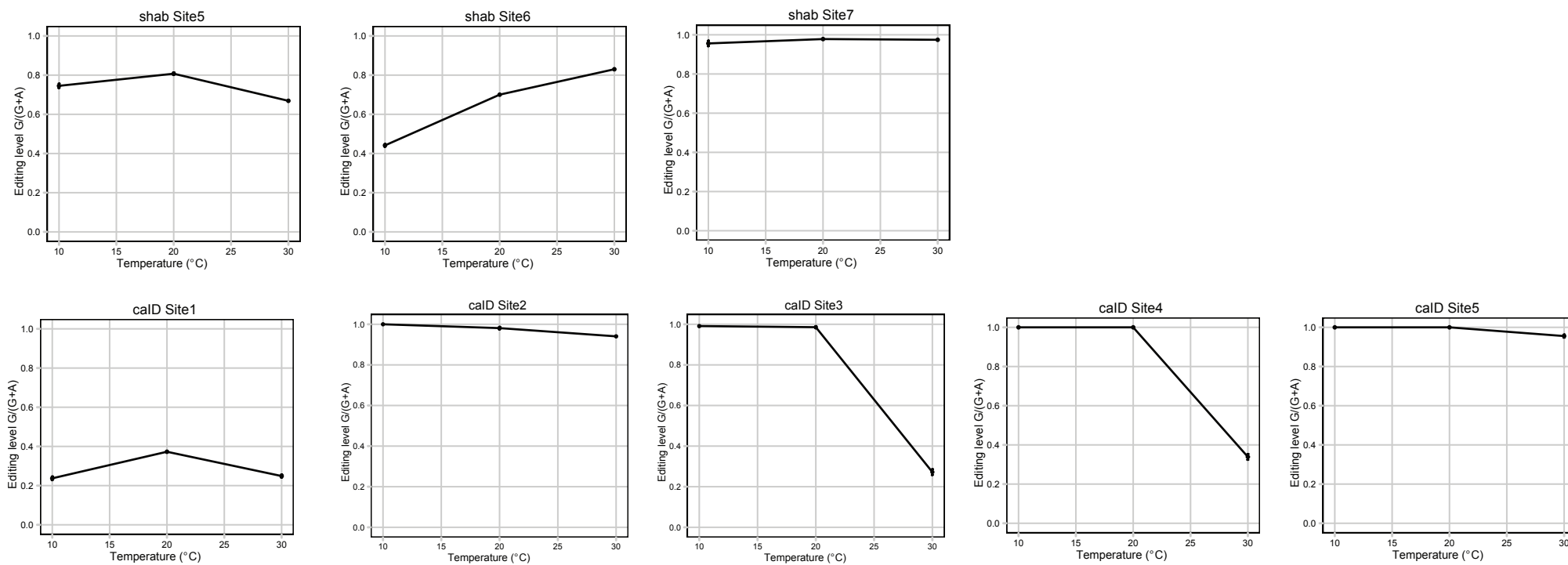

Supplement: Additional file 4: Figure S3. — Examples of different temperature patterns in sites within the same transcripts. (A) The shab transcript contains three editing sites, which respond differently to temperature. (B) The Ca-alpha1D (ca1D) transcript is edited at five sites, each of which displays a different editing pattern in response to temperature. [file 12915_2014_111_MOESM4_ESM.pdf]

**A. Complexin (*cpx*) sites 1-3**

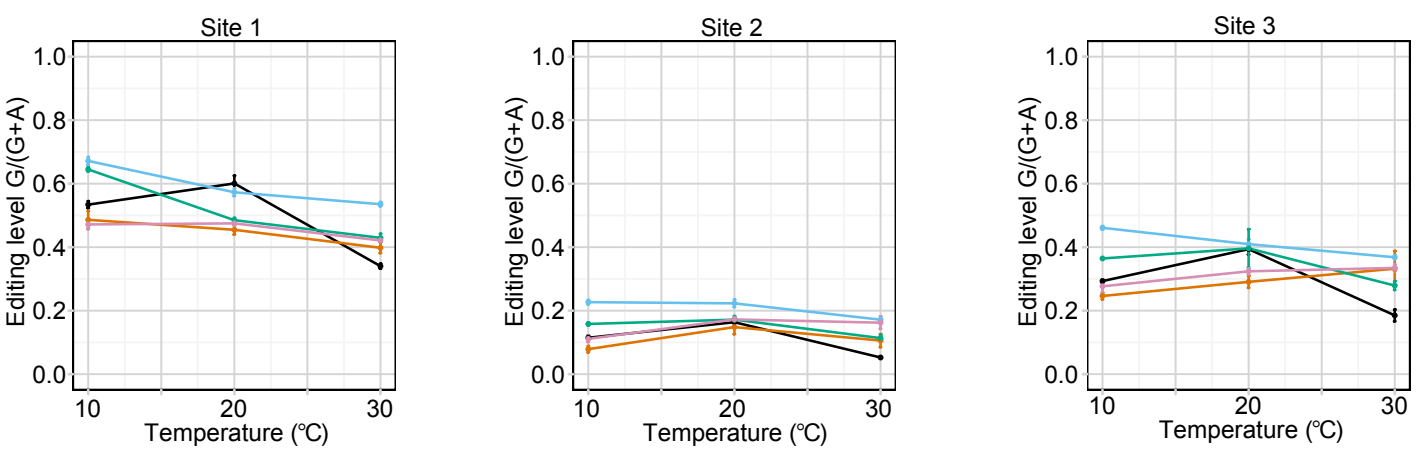

**B. Dadar (*adr*)**

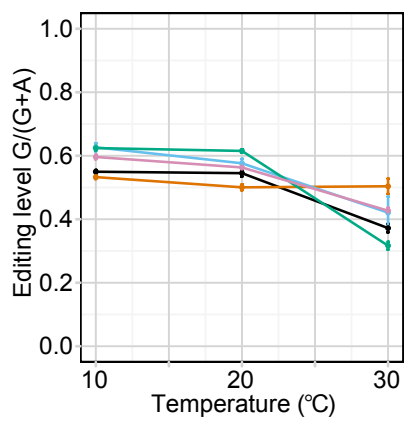

**C. Uncoordinated-13 (*unc*)**

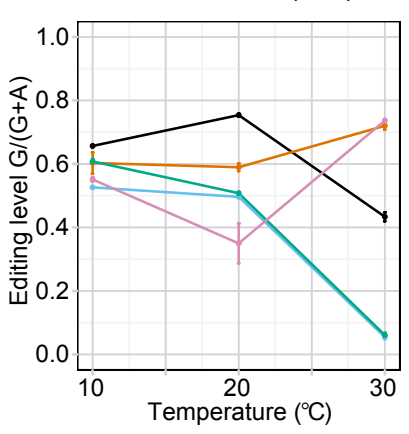

**D**

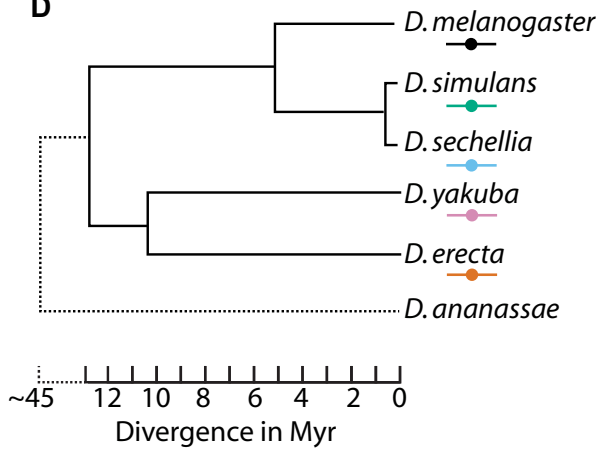

Supplement: Additional file 5: Figure S4. — Conservation of additional editing site responsiveness across Drosophilidae. (A) Complexin sites 1 to 3 are largely temperature unresponsive and these patterns are also conserved across all species studied. (B) Auto-editing of the dadar transcript is stable between 10°C and 20°C, but decreases at 30°C, a pattern that is mostly conserved except in D. erecta. (C) Editing at the single adenosine in the uncoordinated-13 transcript is highly temperature sensitive and does not appear to be conserved between Drosophilidae species. Bars represent standard error in A through C. The slopes of the species-specific editing response curves, rather than the absolute editing, were statistically compared to those of D. melanogaster (black) for each site. Statistics are presented in Esm 6: Table S2. (D) Phylogeny of Drosophilidae species studied, as in Figure 2D. D. ananassae is presented as an outgroup. [file 12915_2014_111_MOESM5_ESM.pdf]

Bio rep1, Tech rep1

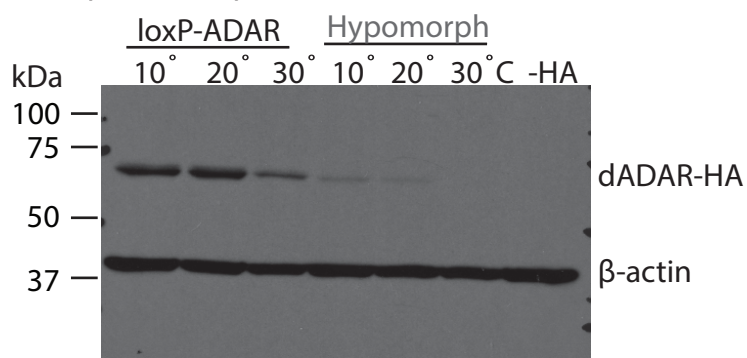

Bio rep1, Tech rep2

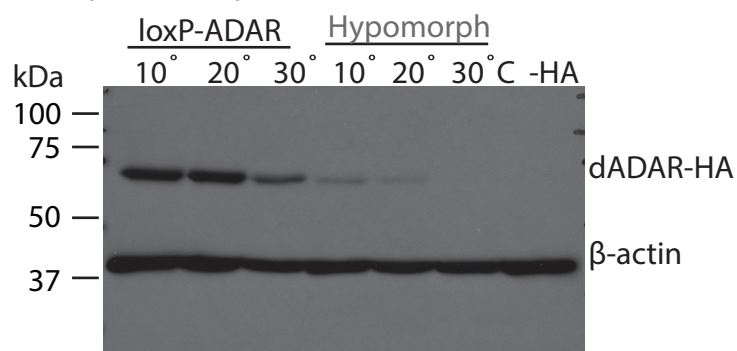

Bio rep2, Tech rep1

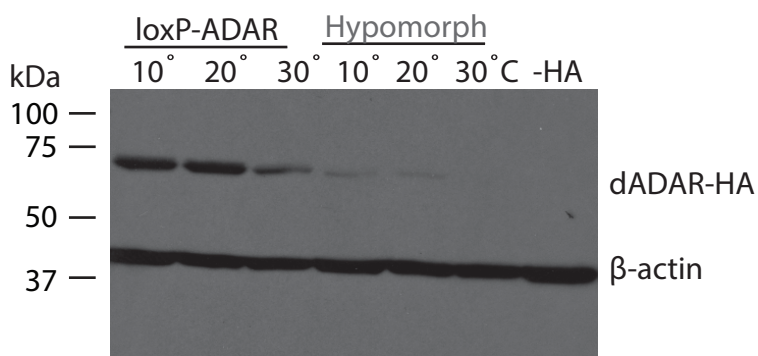

Bio rep2, Tech rep2

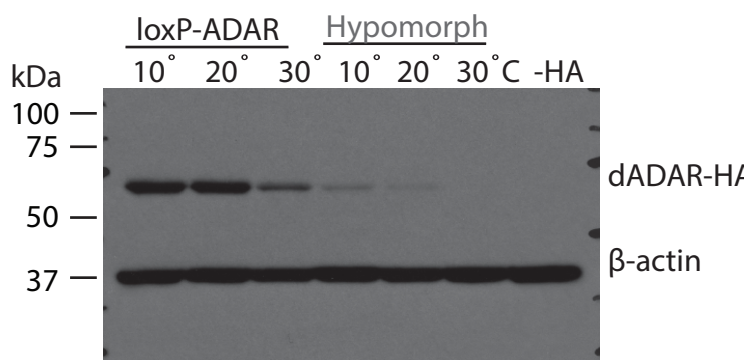

Supplement: Additional file 7: Figure S5. — Raw western blot data used for quantification in Figure 3B. Western blot analysis of the HA-tagged dADAR, as well as the dADAR hypomorph, both generated through homologous recombination [31]. β-actin is presented as a loading control. Three biological and two technical replicates are shown and indicated. [file 12915_2014_111_MOESM7_ESM.pdf]

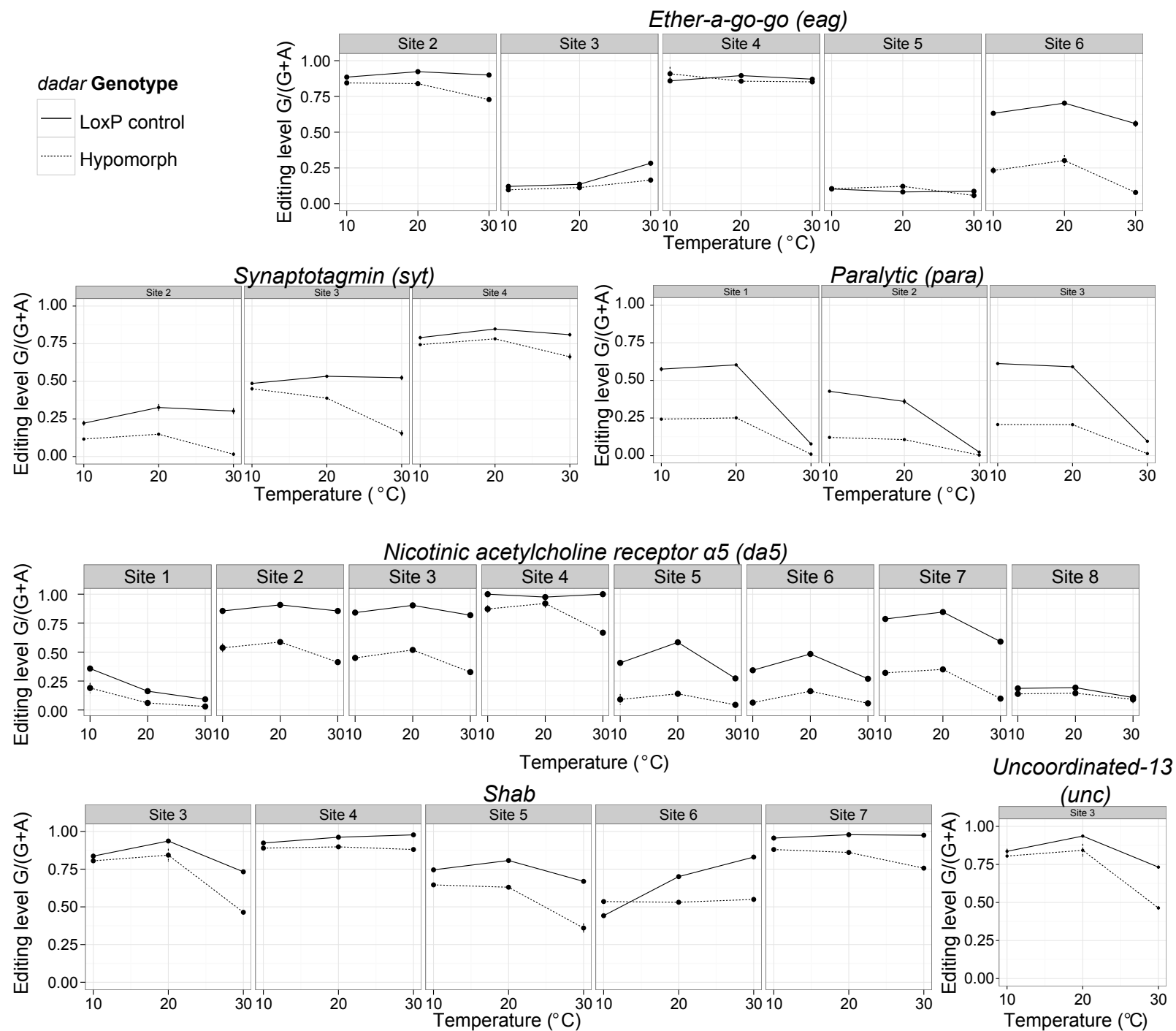

Supplement: Additional file 8: Figure S6. — Editing sites respond differently to temperature in the dADAR hypomorph. Editing is presented for six sites in the ether-a-go-go transcript, three sites in synaptotagmin-1, three sites in paralytic, eight sites in the nicotinic acetylcholine receptor α5 transcript, five sites in shab, and a single site in the uncoordinated-13 transcript. Solid lines represent the editing pattern in the loxP dADAR control, while dotted lines represent the pattern due to the hypomorphic dadar allele [31]. The editing pattern of some sites, for example ether-a-go-go site 4 and shab site 4, changes very little due to decreased levels of dADAR. Other sites, for example ether-a-go-go site 6 and nicotinic acetylcholine receptor α5 sites 2 and 3, change in scale due to decreased dADAR enzyme. Still other sites, including the single site in uncoordinated-13 and shab site 6, display a different temperature responsive pattern in the hypomorph compared to the control. [file 12915_2014_111_MOESM8_ESM.pdf]

# Global Editing as a Function of Temperature and Allele

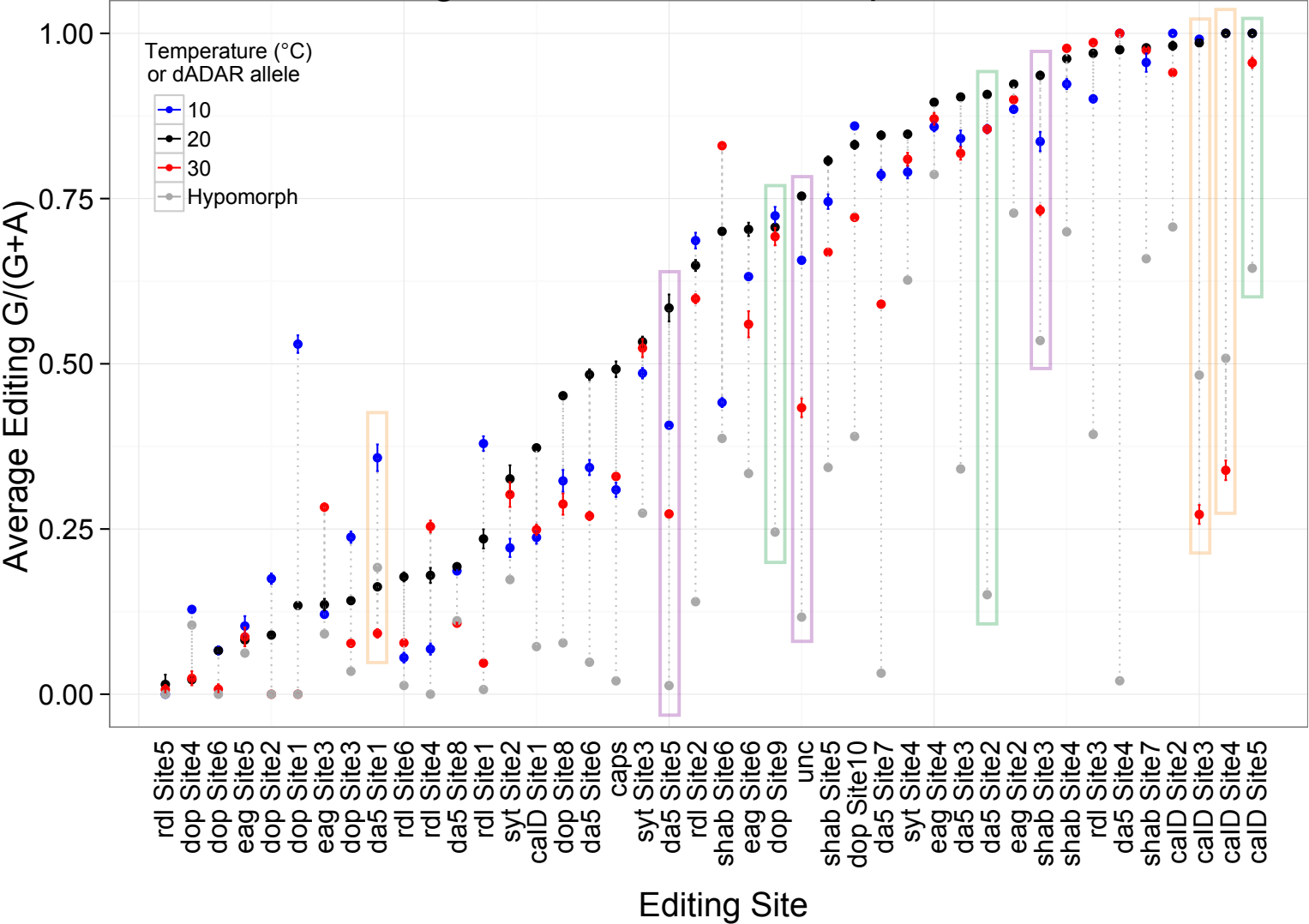

Supplement: Additional file 9: Figure S7. — Editing responsiveness to temperature and dADAR level. The hypomorphic dadar allele results in decreased dADAR protein levels. Here, editing at specific sites in the hypomorph ([32]; gray) is overlaid with the temperature data presented in Figure 1A. A subset of sites is plotted, as not all sites from the current study were assayed in Savva et al. While editing at most sites decreases in the hypomorph, even compared to editing in the wild type animal at 30°C, each site responds differently to temperature changes and dADAR concentration. These data suggest that while decreasing dADAR protein may account for some decrease in editing at 30°C, there is some other factor, for example RNA structure, that is also affected by temperature and impacts editing level. Highlighted sites are reproduced in Figure 3C. [file 12915_2014_111_MOESM9_ESM.pdf]

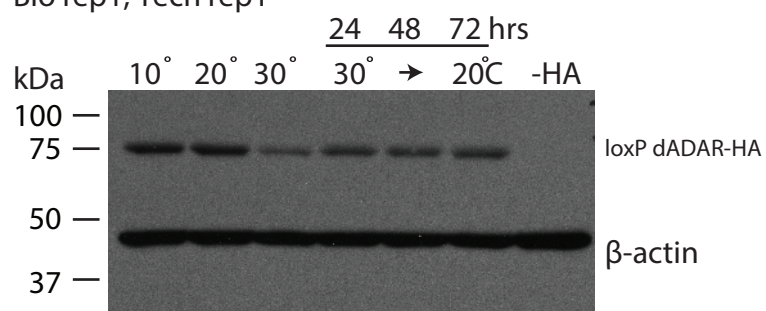

Bio rep1, Tech rep2

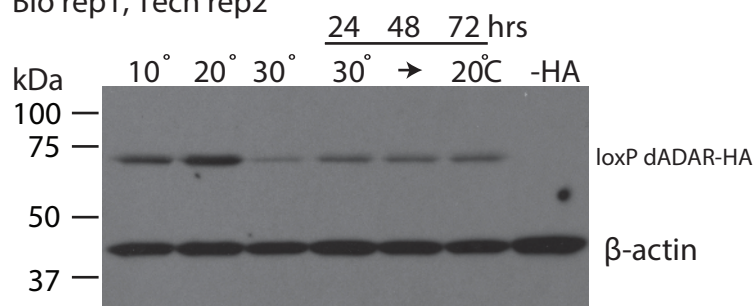

Bio rep2, Tech rep1

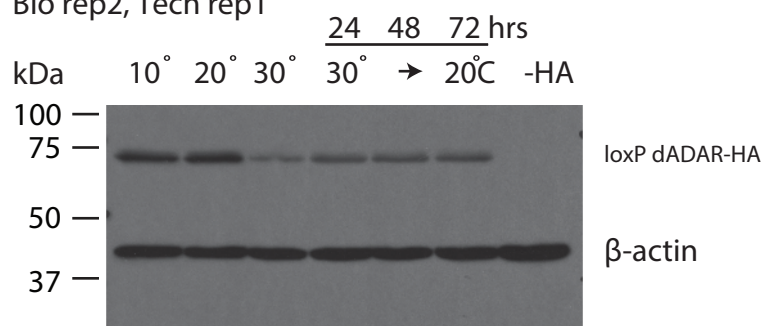

Supplement: Additional file 10: Figure S8. — Raw western blot data used for quantification in Figure 4B. Western blot analysis of the HA-tagged dADAR and the β-actin control after temperature shift and recovery. Three biological replicates and technical replicates are presented and labeled. [file 12915_2014_111_MOESM10_ESM.pdf]

Bio rep1, Tech rep1

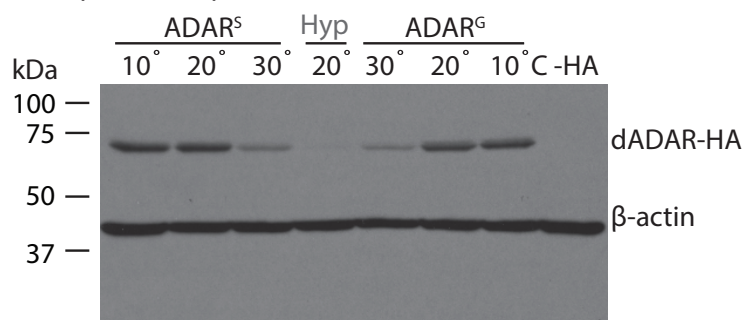

Bio rep1, Tech rep2

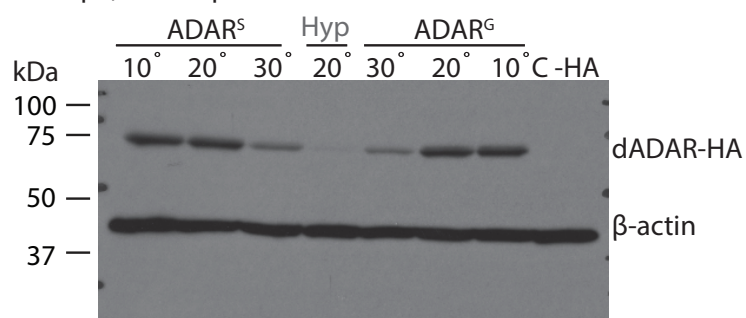

Bio rep2, Tech rep1

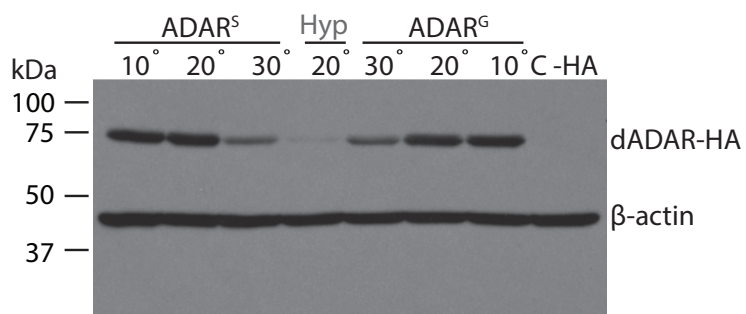

Bio rep2, Tech rep2

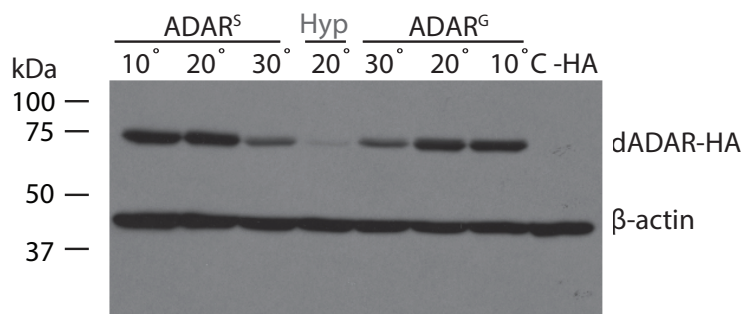

Supplement: Additional file 11: Figure S9. — Raw western blot data used for quantification in Figure 5B. Western blot analysis of the HA-tagged dADARS and dADARG isoforms of dADAR [9] across temperatures. Protein levels from the hypomorphic allele (Hyp) at 20°C are presented for comparison. Wild type dADAR, which lacks the HA tag is presented as a negative control (−HA). β-actin is presented as a loading control. Two biological replicates and two technical replicates are presented and labeled. [file 12915_2014_111_MOESM11_ESM.pdf]

**A. LoxP control**

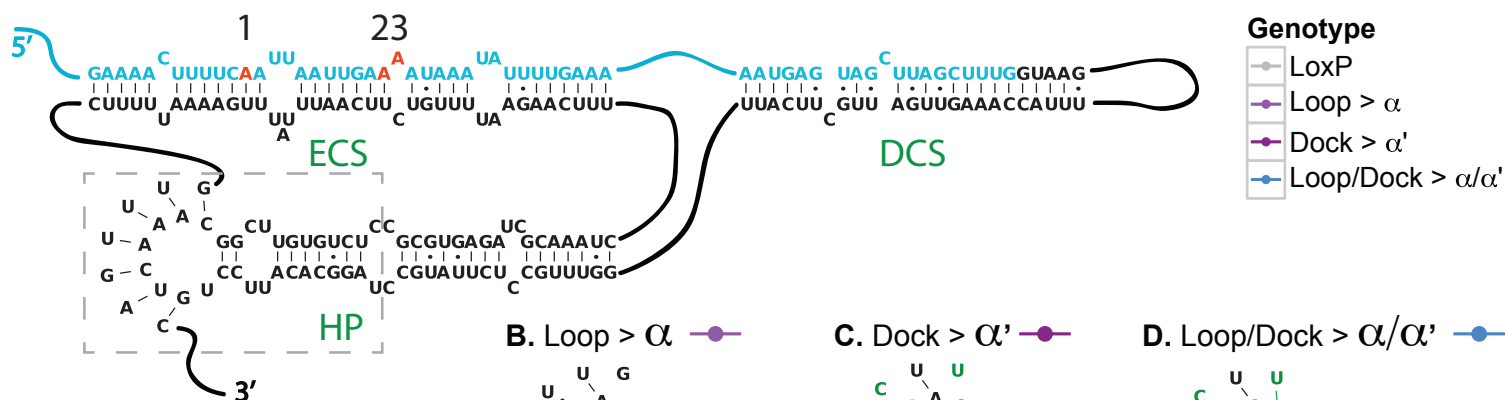

**B. Loop >  $\alpha$**

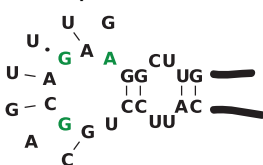

**C. Dock >  $\alpha'$**

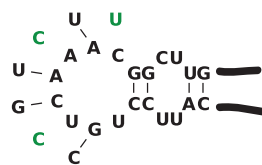

**D. Loop/Dock >  $\alpha/\alpha'$**

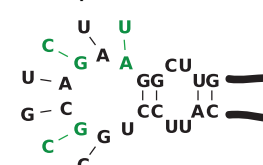

**E.**

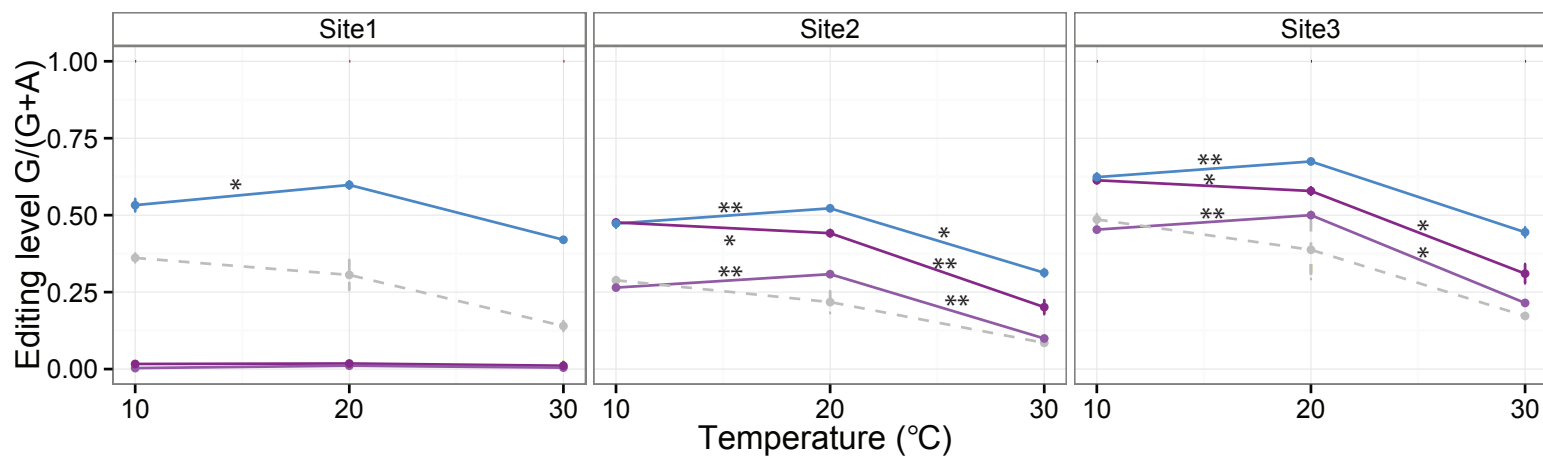

Supplement: Additional file 13: Figure S10. — Effect of tertiary RNA structural mutations on temperature-sensitivity. (A) Paralytic editing sites 1 to 3 (red), within an exon (blue), are encompassed within a complex tertiary structure involving three intronic (black) sequences: the editing site complementary sequence (ECS), the donor site complementary sequence (DCS) and a hairpin (HP), the loop of which forms a tertiary pseudoknot with a docking site upstream of the ECS. The pseudoknot region is boxed for comparison with structural mutations. (B) In the ‘Loop > α’ mutation, the loop region of the hairpin (HP) is mutated at three nucleotides (green), resulting in a loss of tertiary structure and editing at site 1. (C) In the ‘Dock > α’ ‘ mutation, the docking site region is mutated at three nucleotides (green), resulting in a loss of secondary structure, as well as editing at site 1. (D) In the ‘Loop/Dock > α/α’ ‘ mutation, the previous two mutations are combined (green) to restore the tertiary structure and site 1 editing. (E) Because these mutations themselves may result in an overall increase or decrease in the absolute level of editing at all three sites [8], the slopes of the editing response curves, rather than the absolute editing, were compared to that of loxP. The Loop > α (light purple) and Dock > α’ mutation (dark purple) abolish editing at site 1, as shown previously [8], and confer a different editing pattern in response to temperature on sites 2 and 3, compared to the loxP control (gray). The rescue mutation, Loop/Dock > α/α’ (blue) increases editing at all three sites, as shown previously, but also alters the temperature-sensitive response editing pattern in all three sites compared to that of the loxP control (gray). P <0.0001: **, P <0.05: *, P <0.0001: **, P <0.05: *. [file 12915_2014_111_MOESM13_ESM.pdf]

**A. *Synaptotagmin-1 (syt)***

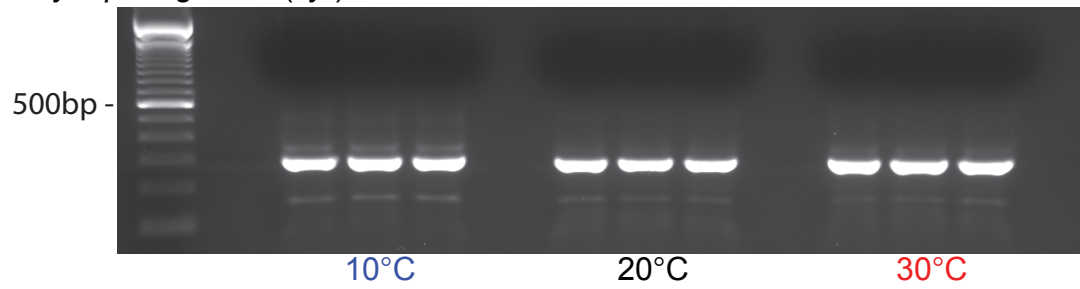

**B. *Paralytic (para)***

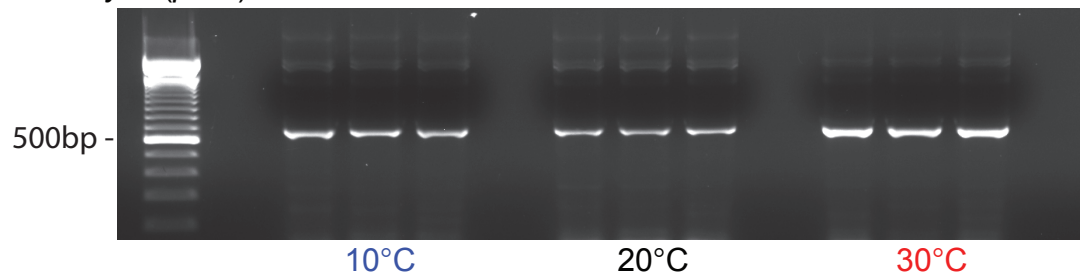

**C. *Shab***

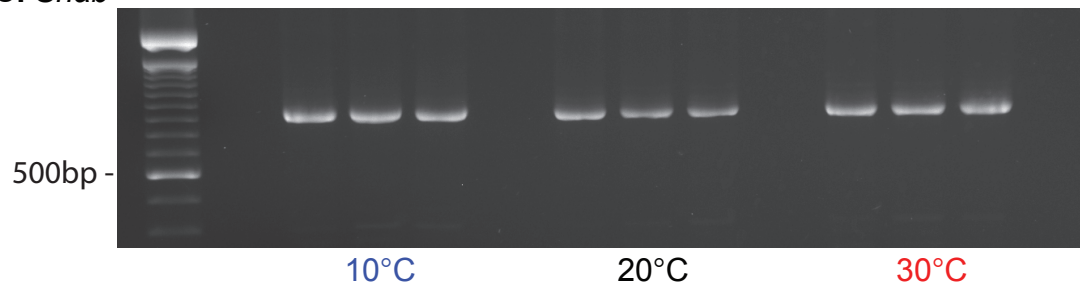

Supplement: Additional file 14: Figure S11. — Representative PCRs from cDNAs showing no change in splicing. PCR products were designed to span exon-exon boundaries surrounding the editing sites. Three replicate PCRs from animals held at each temperature from (A) synaptotagmin-1, (B) paralytic and (C) shab show no splicing change in the vicinity of the editing sites. Lane 1 in each gel shows a 100-bp ladder. This was representative of all the PCR reactions conducted in this study. [file 12915_2014_111_MOESM14_ESM.pdf]

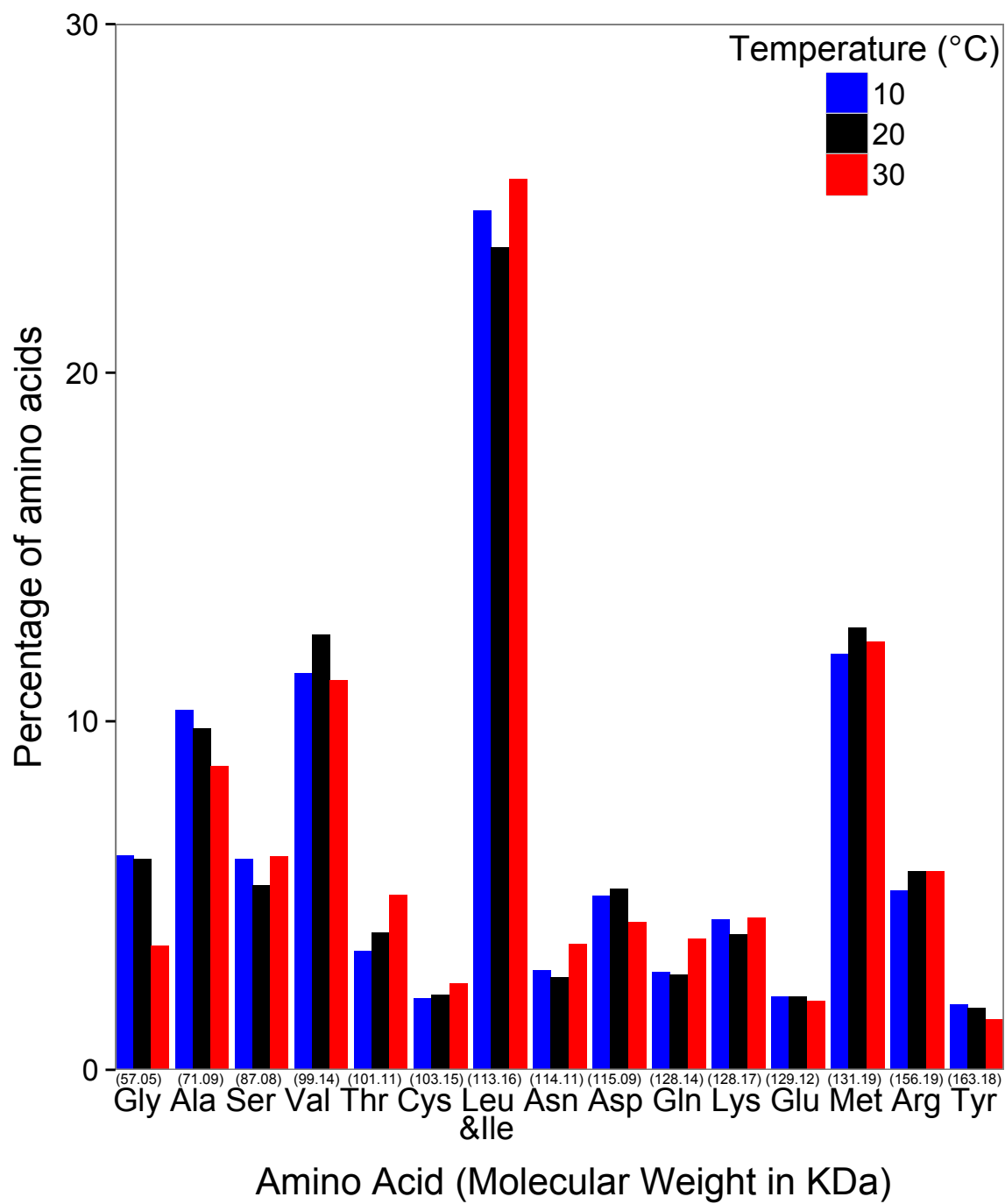

Supplement: Additional file 15: Figure S12. — Relative proportion of amino acids from targeted codons at each temperature. The total proportion of each amino acid in all transcripts, edited and unedited, is presented for 10°C (blue), 20°C (black) and 30°C (red). The codons for 17 amino acids (and all three stop codons) contain at least one adenosine. The only amino acid not represented in our analysis is tryptophan. Editing sites that introduce stop codons were discarded for this analysis. Note that the isomers leucine and isoleucine are combined. The molecular weight of each amino acid is presented in KDa in parentheses. [file 12915_2014_111_MOESM15_ESM.pdf]
