# Supplementary material for: Dynamic response of RNA editing to temperature in Drosophila
Source: BMC Biol. 2015 Jan 3;13:1. doi: 10.1186/s12915-014-0111-3 (PMC4299485; doi:10.1186/s12915-014-0111-3)
Supplement: Additional file 3: Table S1. — Raw data for all temperatures and editing sites. Red sites are highly edited while those in green are edited at a very low level. Standard error is presented for each site at all temperatures. [file 12915_2014_111_MOESM3_ESM.pdf]

| Editing Site | Amino Acid Change | Editing 10°C | Different from 20°C? P-value | Standard Error 10°C | Editing 20°C | Standard Error 20°C | Editing 30°C | Different from 20°C? P-value | Standard Error 30°C |
|---|---|---|---|---|---|---|---|---|---|
| adar | S>G | 0.549665 | 0.935 | 0.007059 | 0.545110 | 0.013444 | 0.372067 | < .0001 (**) | 0.016540 |
| calD Site1 | L>L | 0.237233 | < .0001 (**) | 0.009473 | 0.372823 | 0.003000 | 0.248833 | < .0001 (**) | 0.007769 |
| calD Site2 | N>D | 1.000000 | 0.0163 (*) | 0.000000 | 0.981133 | 0.006343 | 0.940610 | 0.0001 (*) | 0.002874 |
| calD Site3 | L>L | 0.991125 | 0.8749 | 0.003020 | 0.985658 | 0.005091 | 0.272010 | < .0001 (**) | 0.014285 |
| calD Site4 | R>G | 1.000000 | ~ | 0.000000 | 1.000000 | 0.000000 | 0.338808 | < .0001 (**) | 0.014898 |
| calD Site5 | I>M | 1.000000 | ~ | 0.000000 | 1.000000 | 0.000000 | 0.955468 | 0.0001 (*) | 0.007825 |
| caps | M>V | 0.309370 | < .0001 (**) | 0.010776 | 0.491833 | 0.011842 | 0.329465 | < .0001 (**) | 0.004995 |
| cpx Site1 | I>M | 0.534008 | 0.0227 (*) | 0.010918 | 0.600728 | 0.024374 | 0.340290 | < .0001 (**) | 0.007756 |
| cpx Site2 | N>D, G, S | 0.114689 | 0.0053 (*) | 0.007548 | 0.163683 | 0.014128 | 0.052531 | < .0001 (**) | 0.003234 |
| cpx Site3 | N>D, G, S | 0.293040 | 0.0007 (*) | 0.005055 | 0.393768 | 0.017210 | 0.185013 | < .0001 (**) | 0.018437 |
| da5 Site1 | T>A | 0.357793 | < .0001 (**) | 0.020199 | 0.162568 | 0.004385 | 0.092082 | 0.0059 (*) | 0.006539 |
| da5 Site2 | L>L | 0.855310 | 0.0002 (*) | 0.006437 | 0.907578 | 0.003632 | 0.855050 | 0.0002 (*) | 0.006235 |
| da5 Site3 | I>V | 0.840973 | 0.0019 (*) | 0.012122 | 0.903733 | 0.004749 | 0.818390 | 0.0002 (*) | 0.009595 |
| da5 Site4 | T>A | 1.000000 | < .0001 (**) | 0.000000 | 0.975183 | 0.002656 | 1.000000 | < .0001 (**) | 0.000000 |
| da5 Site5 | I>V | 0.407008 | < .0001 (**) | 0.001345 | 0.584423 | 0.020473 | 0.272848 | < .0001 (**) | 0.003531 |
| da5 Site6 | L>L | 0.343043 | < .0001 (**) | 0.011512 | 0.483670 | 0.008016 | 0.269750 | < .0001 (**) | 0.006267 |
| da5 Site7 | I>M | 0.785935 | < .0001 (**) | 0.007740 | 0.845803 | 0.005937 | 0.590265 | < .0001 (**) | 0.002869 |
| da5 Site8 | L>L | 0.186585 | 0.2537 | 0.004012 | 0.193050 | 0.002494 | 0.107455 | < .0001 (**) | 0.001833 |
| da5 Site9 | S>S | 0.901715 | 0.1492 | 0.025103 | 0.859548 | 0.008505 | 0.657590 | < .0001 (**) | 0.004567 |
| dop Site1 | M>S | 0.529770 | < .0001 (**) | 0.013331 | 0.134315 | 0.001866 | 0.000000 | < .0001 (**) | 0.000000 |
| dop Site2 | L>L | 0.174853 | < .0001 (**) | 0.007973 | 0.089606 | 0.004308 | 0.000000 | < .0001 (**) | 0.000000 |
| dop Site3 | S>G | 0.237710 | < .0001 (**) | 0.008660 | 0.141588 | 0.004565 | 0.076920 | < .0001 (**) | 0.001577 |
| dop Site4 | M>V | 0.128338 | < .0001 (**) | 0.004572 | 0.022076 | 0.003727 | 0.024078 | 0.9705 | 0.010563 |
| dop Site5 | L>L | 0.444070 | < .0001 (**) | 0.013282 | 0.337945 | 0.003585 | 0.248388 | < .0001 (**) | 0.004278 |
| dop Site6 | K>E | 0.066300 | 0.9989 | 0.006121 | 0.065970 | 0.004193 | 0.007558 | 0.0002 (*) | 0.007558 |
| dop Site8 | L>L | 0.322855 | 0.0002 (*) | 0.016490 | 0.451705 | 0.004281 | 0.287660 | < .0001 (**) | 0.016115 |
| dop Site9 | I>V | 0.723980 | 0.4687 | 0.013523 | 0.706638 | 0.003705 | 0.692520 | 0.5913 | 0.013271 |
| dop Site10 | STOP>W | 0.859848 | 0.0047 (*) | 0.005096 | 0.831450 | 0.006402 | 0.721498 | < .0001 (**) | 0.001941 |
| eag Site1 | K>R | 0.885048 | < .0001 (**) | 0.002357 | 0.923245 | 0.001705 | 0.899740 | 0.0007 (*) | 0.004349 |
| eag Site3 | Y>C | 0.120850 | 0.296 | 0.003849 | 0.135643 | 0.008714 | 0.283015 | < .0001 (**) | 0.005747 |
| eag Site4 | N>D | 0.858725 | 0.0075 (*) | 0.007099 | 0.895718 | 0.003452 | 0.870675 | 0.0523 | 0.008855 |
| eag Site5 | A>A | 0.103175 | 0.4051 | 0.015052 | 0.082200 | 0.004012 | 0.086708 | 0.9515 | 0.014225 |
| eag Site6 | K>R | 0.631950 | 0.0066 (*) | 0.002395 | 0.703365 | 0.010160 | 0.559813 | < .0001 (**) | 0.019808 |
| para Site1 | Q>R | 0.574788 | 0.0489 (*) | 0.012263 | 0.603035 | 0.003495 | 0.077853 | < .0001 (**) | 0.003272 |
| para Site2 | E>E | 0.427688 | 0.0015 (*) | 0.005495 | 0.359890 | 0.015862 | 0.023668 | < .0001 (**) | 0.001631 |
| para Site3 | N>D | 0.611950 | 0.0429 (*) | 0.006921 | 0.590178 | 0.006666 | 0.095427 | < .0001 (**) | 0.002114 |
| rdl Site1 | L>L | 0.379303 | < .0001 (**) | 0.011081 | 0.235075 | 0.014452 | 0.047065 | < .0001 (**) | 0.003903 |
| rdl Site2 | R>G | 0.686455 | 0.034 (*) | 0.011967 | 0.648798 | 0.008362 | 0.598450 | 0.0075 (*) | 0.006913 |
| rdl Site3 | I>V | 0.900925 | < .0001 (**) | 0.005643 | 0.969828 | 0.002141 | 0.986093 | 0.0206 (*) | 0.001746 |
| rdl Site4 | N>D | 0.068337 | < .0001 (**) | 0.008534 | 0.179903 | 0.011349 | 0.253795 | 0.0008 (*) | 0.009036 |
| rdl Site5 | Q>Q | 0.000000 | 0.7636 | 0.000000 | 0.014831 | 0.014813 | 0.007077 | 0.8573 | 0.007077 |
| rdl Site6 | M>V | 0.055320 | < .0001 (**) | 0.007584 | 0.177588 | 0.006822 | 0.077678 | < .0001 (**) | 0.002664 |
| sbd Site1 | T>A | 0.452693 | < .0001 (**) | 0.012024 | 0.243675 | 0.008129 | 0.308868 | 0.0008 (*) | 0.002057 |
| sbd Site2 | L>L | 0.438240 | < .0001 (**) | 0.015300 | 0.251110 | 0.006769 | 0.268070 | 0.4141 | 0.004414 |
| shab Site3 | T>A | 0.836283 | 0.0001 (*) | 0.014548 | 0.936400 | 0.005527 | 0.732340 | < .0001 (**) | 0.007150 |
| shab Site4 | Y>C | 0.923210 | 0.0005 (*) | 0.007666 | 0.961603 | 0.002324 | 0.977340 | 0.0738 | 0.001405 |
| shab Site5 | T>A | 0.745360 | 0.0006 (*) | 0.011127 | 0.807268 | 0.006643 | 0.668873 | < .0001 (**) | 0.003580 |
| shab Site6 | V>V | 0.441410 | < .0001 (**) | 0.006888 | 0.700350 | 0.002017 | 0.830005 | < .0001 (**) | 0.003784 |
| shab Site7 | I>V | 0.955945 | 0.161 | 0.014172 | 0.978208 | 0.002529 | 0.974525 | 0.9331 | 0.002294 |
| stn | T>A | 0.866243 | 0.864 | 0.017713 | 0.874500 | 0.009670 | 0.646270 | < .0001 (**) | 0.009484 |
| syt Site2 | K>R | 0.221473 | 0.0029 (*) | 0.013892 | 0.325963 | 0.020500 | 0.302115 | 0.5765 | 0.018800 |
| syt Site3 | I>V | 0.485738 | 0.0216 (*) | 0.007975 | 0.533233 | 0.007916 | 0.523770 | 0.7874 | 0.013602 |
| syt Site4 | I>M | 0.790057 | 0.0021 (*) | 0.009578 | 0.847488 | 0.004957 | 0.809523 | 0.03 (*) | 0.009810 |
| unc | S>G | 0.656645 | < .0001 (**) | 0.003956 | 0.753738 | 0.004696 | 0.433482 | < .0001 (**) | 0.014084 |

| Key |
|---|
| 1.000000 |
| 0.750000 |
| 0.500000 |
| 0.250000 |
| 0.000000 |
