# Supplementary material for: Dynamic response of RNA editing to temperature in Drosophila
Source: BMC Biol. 2015 Jan 3;13:1. doi: 10.1186/s12915-014-0111-3 (PMC4299485; doi:10.1186/s12915-014-0111-3)
Supplement: Additional file 6: Table S2. — Raw data for all the editing sites surveyed across species. Red sites are highly edited while those in green are edited at a very low level. Standard error is presented for each site at all temperatures. [file 12915_2014_111_MOESM6_ESM.pdf]

| Editing Site | Species | Editing 10°C | Standard Error 10°C | Slope 10-20°C Different from mel? | Editing 20°C | Standard Error 20°C | Editing 30°C | Standard Error 30°C | Slope 20-30°C Different from mel? |
|---|---|---|---|---|---|---|---|---|---|
| adar | *D. melanogaster* | 0.54967 | 0.00576 | ~ | 0.54511 | 0.01098 | 0.37207 | 0.01350 | ~ |
| | *D. erecta* | 0.53272 | 0.00614 | 0.40920 | 0.50050 | 0.00949 | 0.54310 | 0.02415 | <0.0001 (**) |
| | *D. sechellia* | 0.63242 | 0.00834 | <0.0001 (**) | 0.57624 | 0.01410 | 0.34728 | 0.02153 | <0.0001 (**) |
| | *D. simulans* | 0.62415 | 0.00774 | 0.26540 | 0.61518 | 0.00472 | 0.31674 | 0.01236 | <0.0001 (**) |
| | *D. yakuba* | 0.59612 | 0.00717 | 0.0014 (*) | 0.56312 | 0.01630 | 0.42687 | 0.00949 | 0.0031 (*) |
| cpx Site1 | *D. melanogaster* | 0.53401 | 0.01092 | ~ | 0.60073 | 0.02437 | 0.34029 | 0.00776 | ~ |
| | *D. erecta* | 0.48615 | 0.02787 | <0.0001 (**) | 0.45475 | 0.01502 | 0.39807 | 0.01671 | <0.0001 (**) |
| | *D. sechellia* | 0.67171 | 0.01116 | <0.0001 (**) | 0.57289 | 0.01182 | 0.53536 | 0.00685 | <0.0001 (**) |
| | *D. simulans* | 0.64488 | 0.00630 | <0.0001 (**) | 0.48509 | 0.00722 | 0.42739 | 0.01280 | <0.0001 (**) |
| | *D. yakuba* | 0.47162 | 0.01481 | <0.0001 (**) | 0.47495 | 0.00693 | 0.42117 | 0.00325 | <0.0001 (**) |
| cpx Site2 | *D. melanogaster* | 0.11469 | 0.00755 | ~ | 0.16368 | 0.01413 | 0.05253 | 0.02627 | ~ |
| | *D. erecta* | 0.07880 | 0.01004 | 0.19260 | 0.14825 | 0.02224 | 0.10587 | 0.02068 | <0.0001 (**) |
| | *D. sechellia* | 0.22686 | 0.00681 | <0.0001 (**) | 0.22310 | 0.01178 | 0.17172 | 0.00345 | <0.0001 (**) |
| | *D. simulans* | 0.15822 | 0.00437 | 0.0095 (*) | 0.17176 | 0.01122 | 0.11756 | 0.00984 | 0.0009 (*) |
| | *D. yakuba* | 0.11113 | 0.01000 | 0.58890 | 0.17237 | 0.00644 | 0.16220 | 0.01985 | <0.0001 (**) |
| cpx Site3 | *D. melanogaster* | 0.29304 | 0.00506 | ~ | 0.39377 | 0.01721 | 0.25913 | 0.05389 | ~ |
| | *D. erecta* | 0.24624 | 0.01062 | 0.0055 (*) | 0.29066 | 0.01887 | 0.33221 | 0.07081 | <0.0001 (**) |
| | *D. sechellia* | 0.46095 | 0.00533 | <0.0001 (**) | 0.40950 | 0.01543 | 0.36812 | 0.00630 | <0.0001 (**) |
| | *D. simulans* | 0.36434 | 0.00174 | 0.0025 (*) | 0.40264 | 0.06045 | 0.27906 | 0.01382 | 0.0021 (*) |
| | *D. yakuba* | 0.27673 | 0.02058 | 0.0089 (*) | 0.32386 | 0.00934 | 0.33458 | 0.01057 | <0.0001 (**) |
| para Site1 | *D. melanogaster* | 0.5747875 | 0.01226318 | ~ | 0.603035 | 0.00349515 | 0.077853 | 0.003272211 | ~ |
| | *D. erecta* | 0.536624 | 0.013958054 | <0.0001 (**) | 0.471194 | 0.00450068 | 0 | 0 | 0.0009 (*) |
| | *D. sechellia* | 0.420196667 | 0.012747327 | <0.0001 (**) | 0.354312 | 0.04116774 | 0.136203333 | 0.01233858 | <0.0001 (**) |
| | *D. simulans* | 0.462646667 | 0.031914545 | 0.0104 (*) | 0.438422 | 0.015277 | 0.16323 | 0.019223242 | <0.0001 (**) |
| | *D. yakuba* | 0.4313725 | 0.003847968 | 0.4042 | 0.434958 | 0.00457307 | 0 | 0 | <0.0001 (**) |
| para Site2 | *D. melanogaster* | 0.4276875 | 0.00549525 | ~ | 0.35989 | 0.0158617 | 0.023668 | 0.001630817 | ~ |
| | *D. erecta* | 0.37919 | 0.005351817 | 0.0018 (*) | 0.261768 | 0.00553301 | 0 | 0 | <0.0001 (**) |
| | *D. sechellia* | 0.286753333 | 0.014587605 | 0.8504 | 0.207006 | 0.03297217 | 0.043048 | 0.001508603 | <0.0001 (**) |
| | *D. simulans* | 0.402713333 | 0.031186835 | <0.0001 (**) | 0.256912 | 0.00974026 | 0.1165694 | 0.018729042 | <0.0001 (**) |
| | *D. yakuba* | 0.3087425 | 0.017724105 | 0.4779 | 0.22305 | 0.00624494 | 0.0100562 | 0.006167127 | <0.0001 (**) |
| para Site3 | *D. melanogaster* | 0.61195 | 0.00692076 | ~ | 0.5901775 | 0.00666621 | 0.095427 | 0.002113605 | ~ |
| | *D. erecta* | 0.5705 | 0.008258061 | <0.0001 (**) | 0.467476 | 0.00530796 | 0.035021 | 0.003922198 | <0.0001 (**) |
| | *D. sechellia* | 0.442383333 | 0.00515442 | <0.0001 (**) | 0.348028 | 0.03854251 | 0.124986667 | 0.00511156 | <0.0001 (**) |
| | *D. simulans* | 0.45942 | 0.030718479 | 0.9997 | 0.43537 | 0.01405437 | 0.210172 | 0.014855958 | <0.0001 (**) |
| | *D. yakuba* | 0.451715 | 0.008756283 | 0.7762 | 0.411782 | 0.00224506 | 0.0464202 | 0.003370992 | <0.0001 (**) |
| shab Site6 | *D. melanogaster* | 0.44141 | 0.006887867 | ~ | 0.70035 | 0.002017168 | 0.830005 | 0.003783958 | ~ |
| | *D. erecta* | 0.45919 | 0.001732784 | 0.9836 | 0.707895 | 0.017014952 | 0.848054 | 0.005745507 | 0.96666 |
| | *D. sechellia* | 0.447482 | 0.007588511 | 0.0426 (*) | 0.645112 | 0.004722755 | 0.846138 | 0.005623737 | <0.0001 (**) |
| | *D. simulans* | 0.449712222 | 0.036259615 | <0.0001 (**) | 0.614466667 | 0.020859836 | 0.758831667 | 0.008593839 | 0.5055 |
| | *D. yakuba* | 0.57927 | 0.011190764 | <0.0001 (**) | 0.732412 | 0.021235083 | 0.86914 | 0.006233838 | 0.9401 |
| shab Site7 | *D. melanogaster* | 0.955945 | 0.014171951 | ~ | 0.9782075 | 0.00252916 | 0.974525 | 0.002293936 | ~ |
| | *D. erecta* | 0.930956 | 0.010482725 | 0.6425 | 0.9345 | 0.03150398 | 0.94901 | 0.014107407 | 0.5635 |
| | *D. sechellia* | 0.948716 | 0.013367353 | 0.9889 | 0.976892 | 0.00625312 | 0.977966 | 0.009977499 | 0.9839 |
| | *D. simulans* | 0.920332222 | 0.017619658 | 0.6131 | 0.959075 | 0.01557122 | 0.758831667 | 0.008593839 | <0.0001 (**) |
| | *D. yakuba* | 0.923908 | 0.018170733 | 0.397 | 0.969908 | 0.01282367 | 0.86914 | 0.006233838 | <0.0001 (**) |
| syt Site2 | *D. melanogaster* | 0.22147 | 0.01389 | ~ | 0.32596 | 0.02050 | 0.30212 | 0.01880 | ~ |
| | *D. erecta* | 0.31520 | 0.02655 | <0.0001 (**) | 0.24444 | 0.00920 | 0.09734 | 0.01669 | <0.0001 (**) |
| | *D. sechellia* | 0.34049 | 0.01406 | <0.0001 (**) | 0.31156 | 0.01163 | 0.28656 | 0.01450 | 0.99990 |
| | *D. simulans* | 0.38160 | 0.00642 | <0.0001 (**) | 0.30156 | 0.01566 | 0.17986 | 0.01182 | <0.0001 (**) |
| | *D. yakuba* | 0.28543 | 0.02565 | 0.0438 (*) | 0.35233 | 0.01102 | 0.20710 | 0.00914 | <0.0001 (**) |
| syt Site3 | *D. melanogaster* | 0.48574 | 0.00797 | ~ | 0.53323 | 0.00792 | 0.52377 | 0.01360 | ~ |
| | *D. erecta* | 0.55218 | 0.00932 | <0.0001 (**) | 0.43972 | 0.00549 | 0.33957 | 0.01588 | <0.0001 (**) |
| | *D. sechellia* | 0.51417 | 0.00738 | <0.0001 (**) | 0.47957 | 0.00798 | 0.56638 | 0.00965 | <0.0001 (**) |
| | *D. simulans* | 0.55820 | 0.00650 | <0.0001 (**) | 0.49193 | 0.01033 | 0.44880 | 0.01023 | 0.0004 (*) |
| | *D. yakuba* | 0.47702 | 0.00693 | 0.90180 | 0.51904 | 0.00400 | 0.38913 | 0.00538 | <0.0001 (**) |
| syt Site4 | *D. melanogaster* | 0.79006 | 0.00958 | ~ | 0.84749 | 0.00496 | 0.80952 | 0.00981 | ~ |
| | *D. erecta* | 0.80343 | 0.00958 | <0.0001 (**) | 0.84020 | 0.00271 | 0.77217 | 0.00650 | <0.0001 (**) |
| | *D. sechellia* | 0.77690 | 0.00385 | <0.0001 (**) | 0.79669 | 0.00594 | 0.81588 | 0.00700 | <0.0001 (**) |
| | *D. simulans* | 0.82692 | 0.00562 | <0.0001 (**) | 0.82416 | 0.00767 | 0.80000 | 0.01822 | 0.16920 |
| | *D. yakuba* | 0.78644 | 0.01309 | 0.98750 | 0.84078 | 0.00453 | 0.76084 | 0.00315 | <0.0001 (**) |
| unc | *D. melanogaster* | 0.65665 | 0.00396 | ~ | 0.75374 | 0.00470 | 0.43348 | 0.01408 | ~ |
| | *D. erecta* | 0.60294 | 0.03345 | <0.0001 (**) | 0.58952 | 0.01209 | 0.72011 | 0.01156 | <0.0001 (**) |
| | *D. sechellia* | 0.52611 | 0.00366 | <0.0001 (**) | 0.49625 | 0.00206 | 0.05450 | 0.00623 | <0.0001 (**) |
| | *D. simulans* | 0.60778 | 0.00746 | <0.0001 (**) | 0.50781 | 0.00389 | 0.06090 | 0.00687 | <0.0001 (**) |
| | *D. yakuba* | 0.55108 | 0.00669 | <0.0001 (**) | 0.34971 | 0.06286 | 0.73658 | 0.00473 | <0.0001 (**) |

| Key |
|---|
| 1.000000 |
| 0.750000 |
| 0.500000 |
| 0.250000 |
| 0.000000 |
