# Supplementary material for: Dynamic response of RNA editing to temperature in Drosophila
Source: BMC Biol. 2015 Jan 3;13:1. doi: 10.1186/s12915-014-0111-3 (PMC4299485; doi:10.1186/s12915-014-0111-3)
Supplement: Additional file 12: Table S3. — Raw data and significance for all paralytic editing sites in engineered mutants. Red sites are highly edited while those in green are edited at a very low level. Standard error is presented for each site and mutation at all temperatures. The slope was calculated for each mutant from 10°C to 20°C and 20°C to 30°C and compared to that of the loxP control. P-values are indicated from one-way ANOVAs (α = 0.05) followed by Dunnett post-hoc tests. [file 12915_2014_111_MOESM12_ESM.pdf]

Editing Site	*paralytic* HR Mutant	Editing 10°C	Different from 20°C? P-value	Standard Error 10°C	Editing 20°C	Standard Error 20°C	Editing 30°C	Different from 20°C? P-value	Standard Error 30°C
para Site1	LoxP Control	0.36110	0.3485	0.01398	0.30587	0.04839	0.13976	0.0015 (*)	0.01558
	DCS delete	0.18962	0.883	0.00985	0.18421	0.00983	0.01752	< .0001 (**)	0.00983
	DCS zip	0.60686	0.8407	0.01816	0.58935	0.00821	0.55969	0.5934	0.02487
	HP > α	0.00323	0.2157	0.00253	0.01092	0.00431	0.00462	0.3366	0.00300
	ECS > α'	0.01655	0.9847	0.00307	0.01807	0.00526	0.01080	0.713	0.01080
	HP/ECS > α/α'	0.53255	0.0235 (*)	0.01983	0.59817	0.01161	0.42008	0.0001 (*)	0.00288
para Site2	LoxP Control	0.28856	0.0671	0.00999	0.21756	0.03504	0.08506	0.0025 (*)	0.00451
	DCS delete	0.18322	0.5988	0.01521	0.20506	0.03136	0.02760	< .0001 (**)	0.00487
	DCS zip	0.62686	0.5104	0.00758	0.60431	0.01294	0.43827	< .0001 (**)	0.01590
	HP > α	0.26466	0.001 (*)	0.00756	0.30831	0.00785	0.09939	< .0001 (**)	0.00551
	ECS > α'	0.47635	0.2022	0.00675	0.44150	0.00990	0.20123	< .0001 (**)	0.02236
	HP/ECS > α/α'	0.47281	0.0286 (*)	0.01315	0.52226	0.00500	0.31288	< .0001 (**)	0.01172
para Site3	LoxP Control	0.48616	0.0982	0.00982	0.38758	0.05470	0.17251	0.0014 (*)	0.00271
	DCS delete	0.33500	0.917	0.02052	0.32716	0.01434	0.07995	< .0001 (**)	0.00581
	DCS zip	0.79443	0.1686	0.01112	0.74003	0.01343	0.66182	0.0315 (*)	0.02246
	HP > α	0.45418	0.0007 (*)	0.00783	0.50680	0.00845	0.21491	< .0001 (**)	0.00814
	ECS > α'	0.61342	0.3952	0.00964	0.57855	0.01183	0.31004	< .0001 (**)	0.03078
	HP/ECS > α/α'	0.62363	0.043 (*)	0.01171	0.67452	0.00803	0.44458	< .0001 (**)	0.01535

Key
1.000000
0.750000
0.500000
0.250000
0.000000

Slope (shape)

Editing Site	*paralytic* HR Mutant	Slope 10-20°C Different from Loxp?	Slope 20-30°C Different from Loxp?
para Site1	DCS delete	0.0013 (*)	1.00000
	DCS zip	0.17770	<0.0001 (**)
	HP > α	~	~
	ECS > α'	~	~
	HP/ECS > α/α'	0.0002 (*)	0.99380
para Site2	DCS delete	<0.0001 (**)	0.0047 (*)
	DCS zip	0.0152 (*)	0.12730
	HP > α	<0.0001 (**)	<0.0001 (**)
	ECS > α'	0.006 (*)	<0.0001 (**)
	HP/ECS > α/α'	<0.0001 (**)	0.0118 (*)
para Site3	DCS delete	<0.0001 (**)	0.25010
	DCS zip	0.16790	<0.0001 (**)
	HP > α	<0.0001 (**)	0.0007 (*)
	ECS > α'	0.0002 (*)	0.0068 (*)
	HP/ECS > α/α'	<0.0001 (**)	0.98970
